# Multi-Modality Imaging of the Tricuspid Valve: From Tricuspid Valve Disease to Catheter-Based Interventions

**DOI:** 10.31083/j.rcm2306199

**Published:** 2022-05-30

**Authors:** Fabio Fazzari, Francesco Cannata, Matteo Maurina, Renato Maria Bragato, Marco Francone

**Affiliations:** ^1^Department of Biomedical Sciences, Humanitas University, 20090 Pieve Emanuele, Milan, Italy; ^2^Department of Cardiovascular Medicine, IRCCS Humanitas Research Hospital, 20089 Rozzano, Milan, Italy; ^3^Department of Radiology, IRCCS Humanitas Research Hospital, 20089 Rozzano, Milan Italy

**Keywords:** tricuspid valve intervention, tricuspid regurgitation, echocardiography, computed tomography, cardiac magnetic resonance, multimodality imaging

## Abstract

Tricuspid valve disease represents a major health problem that affects a wide proportion of heart failure patients with a significant prognostic impact. In recent years an increasing number of minimally invasive and transcatheter treatments have been developed. The choice of the optimal transcatheter device therapy needs a careful patient selection and a dedicated anatomic assessment, mainly based on echocardiographic and computed tomography evaluation. Moreover, cardiac magnetic resonance has an established role in the functional assessment of right heart chambers with relevant prognostic implications. In this review we describe the role of multimodality imaging in the tricuspid valve disease assessment with an intervention-oriented perspective, from the pre-operative planning for different devices to the intraprocedural guide during transcatheter edge-to-edge repair.

## 1. Tricuspid Valve Anatomy and Tricuspid Regurgitation Classification

The tricuspid valve (TV) is a complex structure with high inter-individual variations. Its principal components are the leaflets, the papillary muscles with their chordal attachments to the leaflets, and the annulus.

The TV is normally composed of three leaflets. With respect to the orifice area, they are situated anteriorly (anterior or infundibular leaflet), infero-posteriorly (posterior leaflet) and medially (septal or medial leaflet). The anterior and the posterior leaflets are generally the largest and the smallest ones, respectively [1], and the tricuspid orifice area normally ranges between 7 and 9 ${\mathrm{cm}}^{2}$ [2], making it the largest of the four cardiac valves. Compared to the mitral valve, the tricuspid valve leaflets are much thinner, which makes them either more difficult to be imaged at echo [3] and, equally, more fragile.

The tricuspid annulus (TA) is a complex three-dimensional (3D) elliptical-shaped non-planar structure. Compared to the mitral annulus it is more dynamic, showing high variability in size and shape depending on the heart cycle and hemodynamic load conditions [4]. Normal tricuspid annular circumference and area are 12 ± 1 cm and 11 ± 2 ${\mathrm{cm}}^{2}$ [4, 5], but since the tricuspid leaflets physiologically have an excess coaptation length of 5 to 10 mm [6] some annular dilatation may occur before significant regurgitation secondary to mal coaptation develops [2].

Despite many interindividual variants, right ventricular papillary muscles can be schematically distinguished into anterior, posterior, and septal. The first one, normally the largest, originates from the right ventricular apex and the moderator band [6, 7, 8] and is of crucial importance when defining the valve morphology as it serves as a reference point for differentiating the anterior and posterior leaflets.

In the past decades various efforts have been made to establish universal criteria to distinguish between supernumerary leaflet and scallops, but no consensus has been achieved [6, 9, 10].

**Fig. 1.**
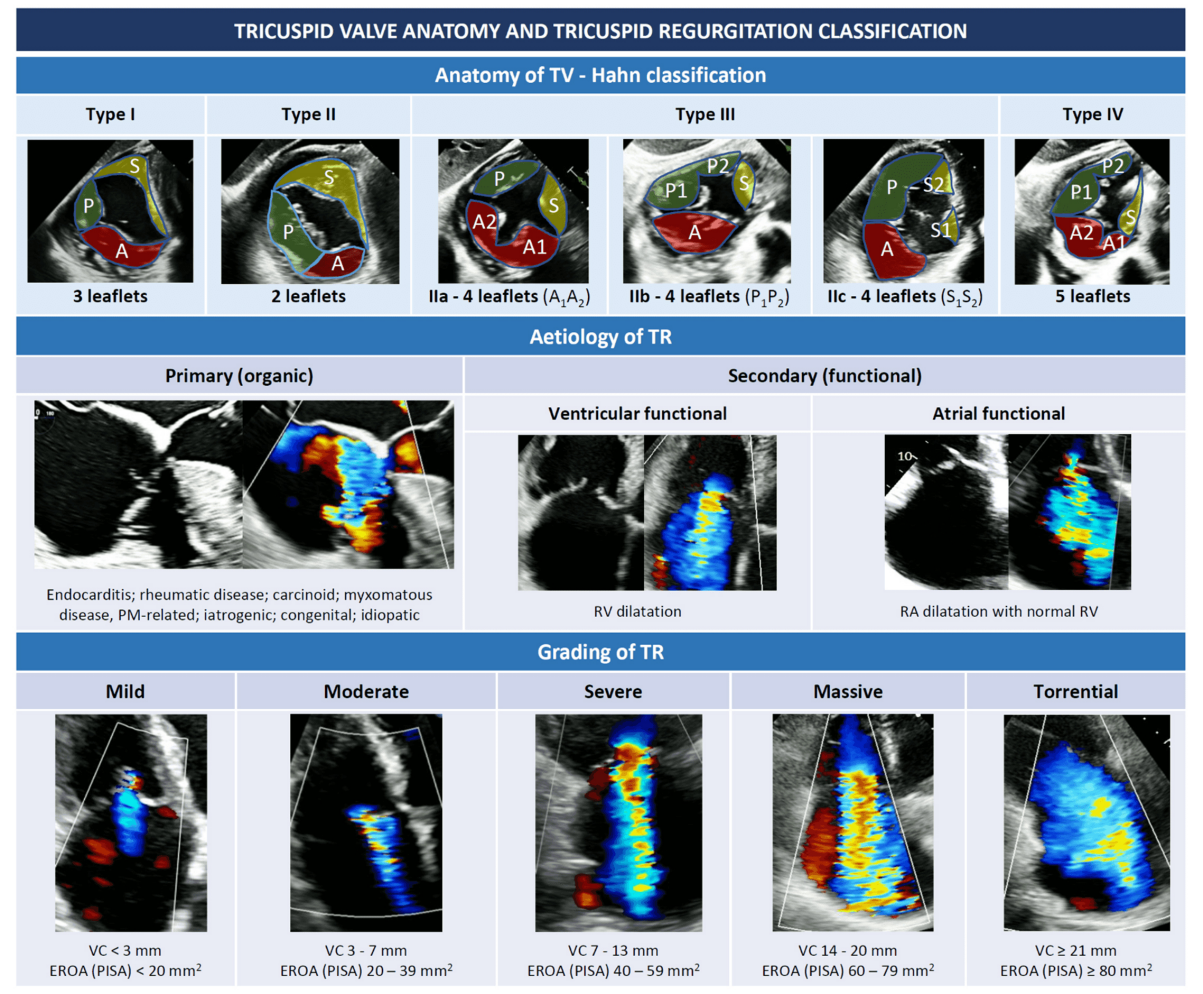
**Tricuspid valve anatomy and tricuspid regurgitation classification (see introduction)**. Abbreviations: EROA, effective regurgitant orifice area; PISA, proximal isovelocity surface area; PM, pace maker; RA, right atrium; RV, right ventricle; TR, tricuspid regurgitation; TV, tricuspid valve; VC, vena contracta.

Finally, the need for an in-depth understanding of the individual tricuspid morphology to guide surgical or percutaneous interventions along with imaging technique innovations and increasing experience, led to the development of a novel universal classification [11]. The Hahn classification (Fig. 1) is currently being adopted as the reference method to classify the tricuspid anatomy [12]. According to the Hahn classification, the TV can be classified into type I (classical three leaflets configuration), type II (2 leaflets with antero-posterior fusion) and type III (4 leaflets with subtypes A, B or C depending on the location of the supernumerary leaflet). Valves with more than 4 leaflets are classified as type IV.

A small degree of tricuspid regurgitation (TR), often referred as “physiological”, is a frequent echocardiographic finding, recognized in up to 80% of healthy individuals [13, 14]. Moderate to severe TR affects approximately 1 in 25 individuals among the elderly, with a higher prevalence in women [13, 15, 16, 17]. While mild or less TR has no impact on long-term survival [18], hemodynamically significant TR has been associated with worse outcomes and an increased risk of morbidity and mortality [18, 19, 20]. Hence, both a correct grading and recognition of the underlying mechanism of TR are crucial before considering any procedure on the TV.

From an etiological point of view, TR can be classified into primary or secondary (Fig. 1).

Primary TR (also called “organic”) is relatively rare and is a consequence of a primitive alteration of the valvular complex, either acquired or congenital. In young individuals, primary TR is more often congenital, with Ebstein anomaly being most common, while acquired TR is rare and generally due to trauma or infective endocarditis in drug users. In the overall population, the most frequent causes of primary TR are endocarditis, rheumatic disease, carcinoid, myxomatous disease, endomyocardial fibrosis and iatrogenic damages. These latter include cardiac implantable electronic device (CIED) lead-related injuries to TV due to impingement, foreign body inflammation and leaflets fibrosis, endocarditis, or even direct leaflets laceration [21, 22, 23].

Secondary TR (or “functional”) is by far the most frequent type of TR, representing approximately 90% of all TR [24], and is due to right ventricle (RV) or TA dilatation without evident alterations of the valvular complex [25]. Both pressure and volume overload may be responsible for RV dilatation, while chronic atrial fibrillation (AF) is the most frequent cause of atrial dilatation and annular enlargement [26].

Ventricular FTR may be due to left-heart disease (either valvular of ventricular dysfunction), pulmonary hypertension (PH), or any type of RV dysfunction (either congenital or not), that cause papillary muscles displacement, leaflets tethering, and finally, as shown by 3D echocardiographic studies, annular deformation [5]. The annular dilatation in FTR predominantly occurs along the antero-lateral side, where cardiac tissue is less resistant, as the septal and the posterior leaflets insert into fibrotic part of the annulus and into the diaphragm-supported inferior RV wall, respectively [17].

In contrast, the so-called “atrial-functional” or “atriogenic” TR occurs because of isolated right atrial dilatation with normal RV, most often in presence of long-standing AF with progressive atrial and annular enlargement [26]. Since the definition of this entity is recent and no dedicated large studies exist, current guidelines do not specifically address atriogenic TR [27, 28] and additional studies are needed to better define this entity and possibly hint at specific therapeutic strategies.

As mentioned before, since high degrees of TR correlate with worse outcomes, classifying TR according to its severity is also important and echocardiography remains the reference imaging technique. Classical grading scheme for TR included mild, moderate or severe grades, with severe TR defined by the presence of a vena contracta (VC) ≥7 mm, an effective regurgitant orifice area (EROA) ≥40 ${\mathrm{mm}}^{2}$, and a regurgitant volume ≥45 mL [14]. However, early insights from the SCOUT trial showed that standard nomenclature fails to take into account the most extreme (or “very severe”) degrees of TR [29, 30]. For this reason, a new 5-class grading scheme including the “massive” and “torrential” grades (Fig. 1) has been proposed [30] and adopted in recent interventional studies evaluating different interventional procedures on the TV [31, 32, 33, 34]. Interestingly, patients with massive or torrential TR are exposed to a higher risk of death and readmission for heart failure (HF) than severe TR [35]. In addition, both baseline massive or torrential TR have shown to be independent predictors for achieving moderate or less TR after transcatheter tricuspid valve intervention (TTVI) [34].

Based on this evidence, this upgraded 5-class severity grading scheme seems to have important prognostic implications, and hopefully will be systematically adopted in future studies addressing the TV.

Echocardiography remains the most used technique to assess for tricuspid anatomy and eventually evaluate the type and grade of TR. However, many other imaging modalities are of undoubtful importance, and their role will be discussed in the following paragraphs.

## 2. Diagnostic Work-Up of Severe Tricuspid Regurgitation

### 2.1 Echocardiography

Echocardiographic imaging is key for TV assessment and the initial tool for the evaluation of the right heart chambers. A comprehensive and multi-modality imaging of the TV and right heart chambers is initially based on trans-thoracic echocardiography (TTE) with additional and complementary aid of trans-esophageal echocardiography (TEE), cardiac computed tomography (CCT) and cardiac magnetic resonance (CMR).

#### 2.1.1 Transthoracic Echocardiography 

Due to its complex nature, the assessment of TV morphology and disease mechanism requires multiple TTE and TEE windows [2].

The main TTE views are the left parasternal long-axis (LAX) view focused on the RV inflow, the left parasternal short-axis (SAX), the apical and the subcostal views (Table 1). The visualization of all leaflets in one two-dimensional (2D) view is only rarely achieved, mainly through modified subcostal or parasternal SAX windows. A precise TTE characterization of the leaflets is challenging but few anatomic landmarks may be helpful to guide the imager (Table 1) [2, 36, 37]. The aortic valve permits to localize the anterior leaflet, in particular the non-coronary cusp is adjacent to the antero-septal commissure. The entry point of coronary sinus into the right atrium (RA) locates the postero-septal commissure, instead.

**Table 1.** **Trans-thoracic views of tricuspid valve and anatomical landmarks for leaflets identification**.

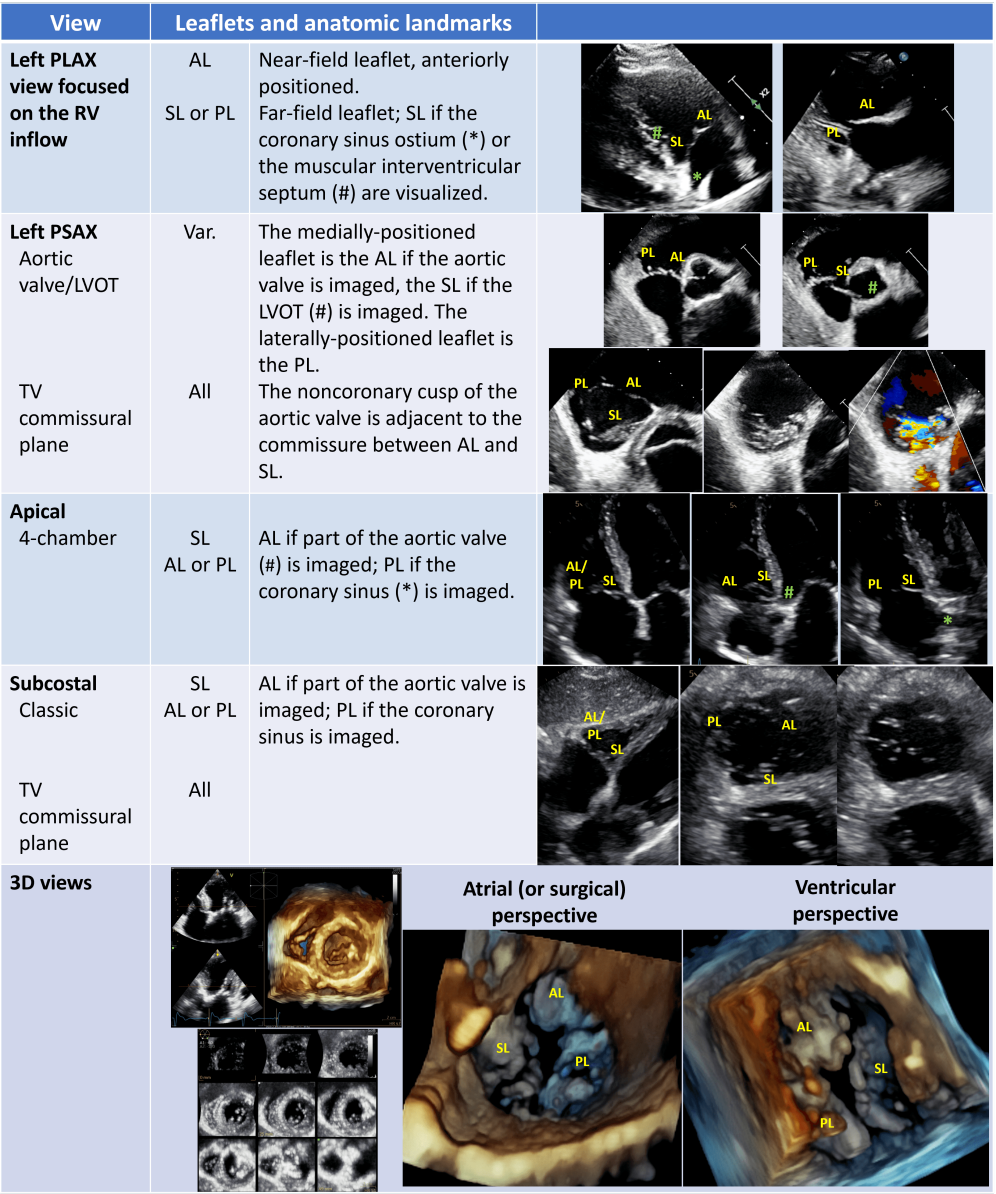

Abbreviations: AL, anterior leaflet; LVOT, left ventricular outflow tract; PL, posterior leaflet; PLAX, parasternal long-axis; PSAX, parasternal short-axis; SL, septal leaflet; TV, tricuspid valve; Var., variable.

#### 2.1.2 Transesophageal Echocardiography 

A comprehensive TEE examination of the TV should include multiple windows from several depths and angles and the recurring use of biplane (or crossplane) modality [2, 38, 39].

The multilevel assessment of the TV classically begins at a mid-esophageal (ME) depth with two views (Table 2): ME 4-chamber view (at about $0^{\circ }$ degrees) which shows the septal leaflet and generally the anterior leaflet, though a retroflexion movement of the probe may reveal the posterior leaflet. The use of biplane mode is useful to clearly define which leaflet is visualized, also based on the above-mentioned anatomical markers (Table 2). The second ME view is the RV inflow-outflow view (at about ${\mathrm{60}}^{\circ }$ degrees), which shows the anterior leaflet (adjacent to the aortic valve) and the posterior leaflet (attached to the posterolateral wall of the RV), while the septal leaflet lies behind on a different imaging plane. If imaged with biplane mode, this view allows to entirely span the coaptation line of the septal leaflet with both the anterior and the posterior leaflets.

**Table 2.** **Trans-esophageal views of tricuspid valve and anatomical landmarks for leaflets identification**.

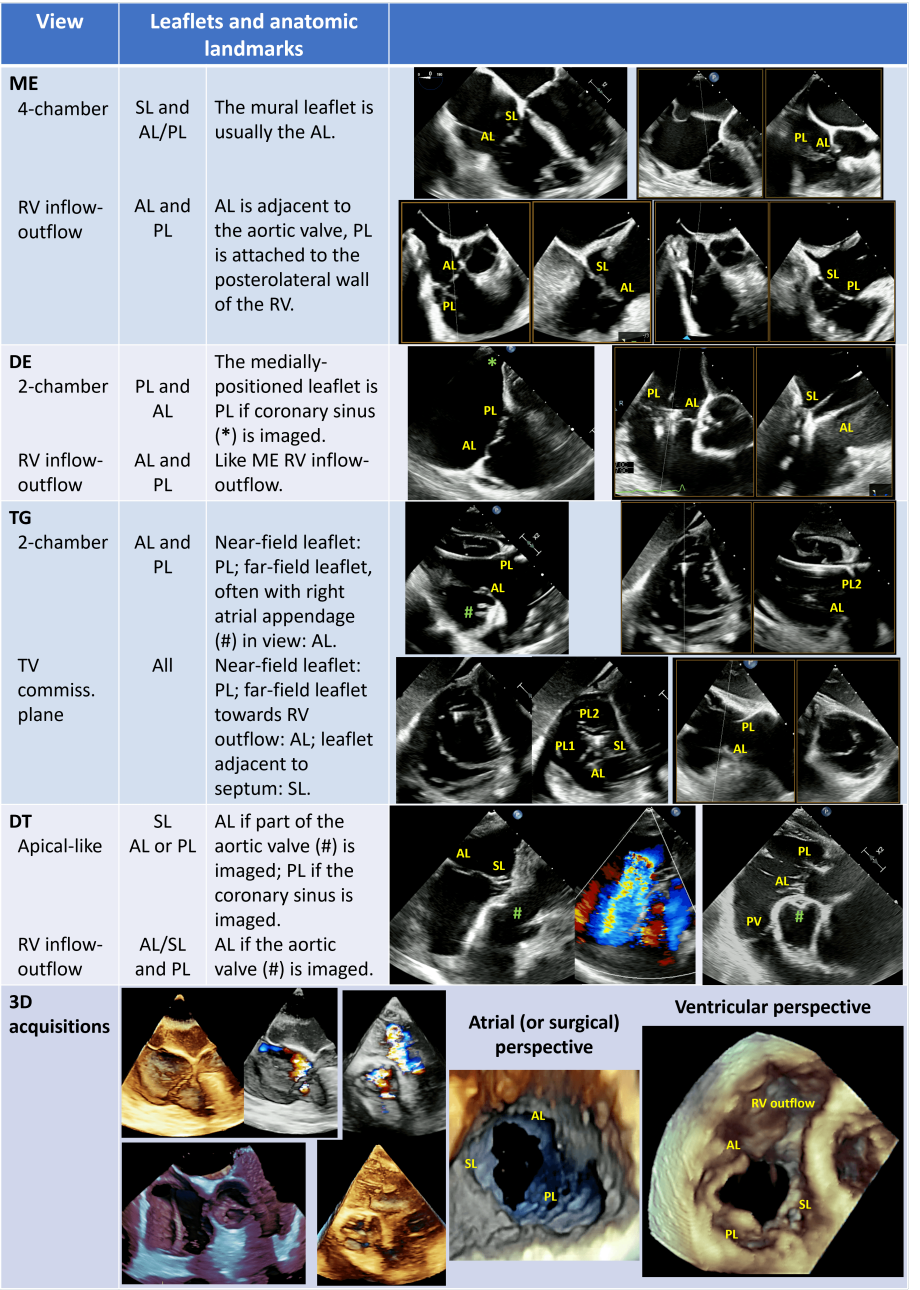

Abbreviations: AL, anterior leaflet; DE, deep-esophageal; DT, deep-transgastric; ME, mid-esophageal; PL, posterior leaflet; PV, pulmonary valve; RV, right ventricle; SL, septal leaflet; TG, transgastric; TV, tricuspid valve.

At deep-esophageal (DE) level the probe becomes closer to the RA and the TV without the interposition of the left atrium. Due to this proximity, DE views are optimal for 3D acquisitions of the TV and for Doppler beam alignment with regurgitant jets. Like at ME level, main DE views are the 4-chamber and the RV inflow-outflow.

At trans-gastric (TG) level unlimited views of the TV may be imaged, with a meticulous manipulation of the probe (usually right and anterior flexion) and use of different angles and biplane mode. The SAX view of the TV is crucial to assess the valve anatomy (number and morphology of leaflets, presence of clefts/fold indentations), to identify the regurgitant orifice and to measure the coaptation gap size. This view is required for assessment of the TV anatomy according to the above-mentioned classification of Hahn *et al*. [11]. If a CIED-lead crosses the TV, this view allows to localize where the lead crosses the valve and to assess the presence and entity of a potential CIED-related interference with leaflet coaptation.

Finally, deep-transgastric (DT) views of the TV may be useful mainly for colour flow evaluation and optimal Doppler beam alignment. In case of intense shadowing of the TV at ME and DE levels due to the interposition of atrial septum, aortic and mitral valves, this window permits to solve this issue.

#### 2.1.3 Three-Dimensional Echocardiography

Given the limitations and challenges of 2D echocardiography for TV assessment, 3D imaging is nowadays recommended to provide an exhaustive evaluation of leaflets, annulus and subvalvular apparatus. 3D datasets may be acquired from any good-quality TTE or TEE view [28]. The TTE apical (RV-focused or foreshortened) and parasternal RV inflow views are generally the best approaches to achieve an optimal TTE 3D acquisition of TV, while the TEE DE view permits to reduce at minimum the distance between the TEE probe and the right heart and to acquire fulfilling 3D datasets [40, 41]. A good 3D acquisition always derives from a 2D view which has been adequately optimized in terms of gain, with a high tissue-blood contrast and low speckle noise. To achieve the best spatial resolution of 3D datasets, it is pivotal to maintain the whole TV within a small acquisition volume, while optimizing the acquisition volume size and shape, and the gain and temporal resolution settings. To comprehensively evaluate the anatomy of the TV, the valve is generally visualized “en face” from both the ventricular and the atrial perspectives. The atrial (or surgical) view is particularly useful when analysing a primary TR, as it allows a detailed assessment of the motion of the leaflets; the ventricular perspective provides information regarding the involvement of commissures or fold indentations into TR mechanism, the presence of a leaflet/chordal impingement due to a CIED or the entity/location of the regurgitant orifice. A multibeat acquisition is generally preferred, as it provides the highest resolution and frame rate. However, in presence of arrhythmias or marked respiratory variations, multibeat acquisitions are prone to stitching artifacts. Real-time or live 3D is particularly useful for the guidance of transcatheter procedures as it is less susceptible to motion artifacts and the use of real-time multiplanar reconstruction (MPR) allows rapid orientation within the 3D dataset. The above-mentioned anatomic landmarks (aortic valve, septum, coronary sinus) for leaflet identification need to be encompassed within the 3D dataset, in order to allow a correct interpretation of the 3D TV acquisition [41].

There is not a general agreement regarding the proper orientation of the en-face view of the 3D TV acquisition. Lang *et al*. [42] proposed to orient the TV with the septal leaflet at 6 o’clock regardless of the atrial or ventricular perspective. More recently, for interventional purposes, Muraru *et al*. [40] proposed an atrial perspective with the septal leaflet between 6 and 10 o’clock, whereas Agricola *et al*. [43] oriented the atrial view with superior vena cava (SVC) at 11 o’clock and inferior vena cava (IVC) at 7 o’clock.

3D echocardiographic imaging cannot always inform regarding the leaflet thickness and tissue features. In particular, the tricuspid leaflets are much thinner than the mitral ones, with a generally poorer echocardiographic definition, which is widely influenced by the leaflet orientation with respect to the ultrasonographic beam: an echocardiographic view with an annular plane perpendicular to the insonation beam usually provides better definition of the leaflets in systole (closed valve), while the reverse occurs for annular planes parallel to the beam [40]. Then, blurring artifacts alter the imager’s perception of leaflet thickness, while 3D traditional colour maps represent a depth map and do not directly mirror the tissue characteristics.

**Fig. 2.**
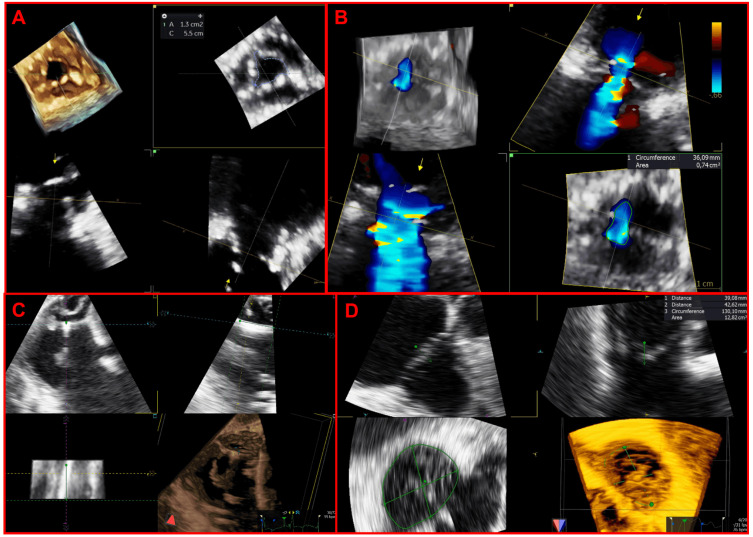
**Applications of multiplanar reconstructions of 3D datasets of the tricuspid valve**. (A) 3D multiplanar reconstruction of a stenotic tricuspid bioprosthetic valve, used to planimeter the residual bioprosthetic orifice area. (B) Multiplanar reconstruction of a 3D color-Doppler volume dataset for quantification of regurgitant vena contracta area. (C) 3D multiplanar reconstruction used to assess the transvalvular trajectory of a CIED-lead during the entire cardiac cycle and its relationships with leaflets and the subvalvular apparatus. (D) 3D multiplanar reconstruction used to planimeter the annular perimeter, area and diameters.

Unlimited useful information may be derived from high-quality 3D datasets thanks to offline MPR, which permit an in-depth exploration of the multiple 2D planes included within the acquired 3D volume at any point in the cardiac cycle (Fig. 2).

#### 2.1.4 Right Ventricle and Right Atrium: Two and Three-Dimensional Echocardiography 

Echocardiography is the first-line and most widely used imaging tool for the assessment of dimensions and function of the right chambers.

The RV anatomy is notoriously complex, with a crescentic shape in the sagittal plane, a triangular profile in SAX view, and three different and indissoluble regions: inflow, apex and outflow [44]. Because of this unique complexity, a standardised approach is needed to achieve a reliable and reproducible echocardiographic assessment of this chamber. In particular, the RV-focused view should always be acquired and analysed as it has shown superior reproducibility for the evaluation of function and size parameters as compared with the standard apical view [45, 46]. Commonly used 2D measurements include basal, middle, and longitudinal dimensions for RV size assessment and tricuspid annular plane excursion (TAPSE), S’, fractional area change, and free-wall longitudinal strain for RV function evaluation. Even if each of these measures shows a significant prognostic meaning, their accuracy and reproducibility are suboptimal. Indeed, TAPSE and S’ assess only the longitudinal excursion of the RV base, whereas more comprehensive parameters such as fractional area change, and free-wall longitudinal strain are based only on a single tomographic plane. Thus, the use of 3D echocardiography is essential for a full and reliable assessment of the complex shape and systolic function of the RV. 3D echocardiography measures RV volumes and ejection fraction (EF) parameters otherwise measured only with CMR and CCT—with an excellent agreement as compared with CMR gold-standard, with a tiny underestimation of RV volumes by 3D echocardiography, but comparable values of RV ejection fraction (RVEF) between the two modalities [47, 48]. RV function assessment in the context of severe TR is even more challenging as all traditional parameters are closely dependent on loading conditions; in this perspective, 3D measurements are even more crucial. Moreover, 3D-derived parameters of RV size and function have been shown to provide additive and incremental prognostic information over other traditional echocardiographic parameters [49].

Differently from the left ventricle, the right one is a volume-loaded pump with a great compliance and a thin myocardial wall. In response to volume or pressure overload the right chambers exhibit different patterns of remodelling and consequently different mechanisms of secondary TR [50]. In patients without left-sided heart disease and without PH, the right atrial enlargement with consequent annular dilatation due to AF or HF with preserved EF is the primary driver of significant secondary TR, whereas the RV dimensions and function are normal (at least during the first stages of the TR pathophysiology) [50, 51]. RV pressure overload due to left-sided heart disease and PH causes various grades of RV dilation and dysfunction with consequent papillary muscle displacement and leaflet tethering-related secondary TR. Similarly, RV dilation and dysfunction due to primary RV disease (ischemic or cardiomyopathy) progressively causes a ventricular FTR [51]. Hence RV dilatation with TV tenting and right atrial dilatation with TA dilatation develop differently based on the specific underlying aetiology leading to FTR. 3D echocardiography, thanks to its volumetric accuracy, inter-operator reproducibility, and new tricuspid-specific quantification tools, is the best modality for an in-depth analysis of the interplay between the complex and long-neglected structures of the right heart [44, 50, 52, 53].

Moreover, due to the known sensitivity of the RV to pressure overload, the concept of RV-pulmonary artery coupling has been recently introduced to correctly assess the RV contractility response to increased afterload [54, 55]. The ratio between non-invasive measures of RV function (usually TAPSE) and echocardiographic-derived systolic pulmonary artery pressure (sPAP) has shown an independent prognostic value in several patient subsets, even those with severe TR [12]. As far as RV and pulmonary vasculature are coupled so that the increase of TAPSE (or any RV function non-invasive measure) and sPAP is comparable with a stable ratio, the RV is able to compensate the pressure overload. When the RV and pulmonary vasculature are not coupled anymore and the TAPSE/sPAP ratio decreases, that is the onset of RV failure. However, the diagnostic sensitivity for PH of echocardiography is limited (55%) and the patient cohort with discordant results from invasive and echocardiographic assessment showed the worst outcomes after transcatheter tricuspid valve repair (TTVR), probably because of a more severe TR degree [56]. Consequently, invasive measurements of pulmonary arterial pressures with right heart catheterization should always be performed in patients with severe TR, above all those screened for transcatheter therapies.

#### 2.1.5 Echocardiographic Differentiation of TR Mechanism

Proper analysis of TR mechanism is the first step for an adequate definition of the patient’s diagnostic and therapeutic pathways.

TR can be divided into primary, secondary and CIED related [12]. The characterization of TR mechanism is based on a comprehensive imaging assessment of three elements: leaflet mobility, annular dimensions, type of RV and RA remodelling.

Table 3 shows a classification of TR mechanisms based on pathophysiology and leaflet mobility.

**Table 3.** **Classification of the mechanisms of tricuspid regurgitation**.

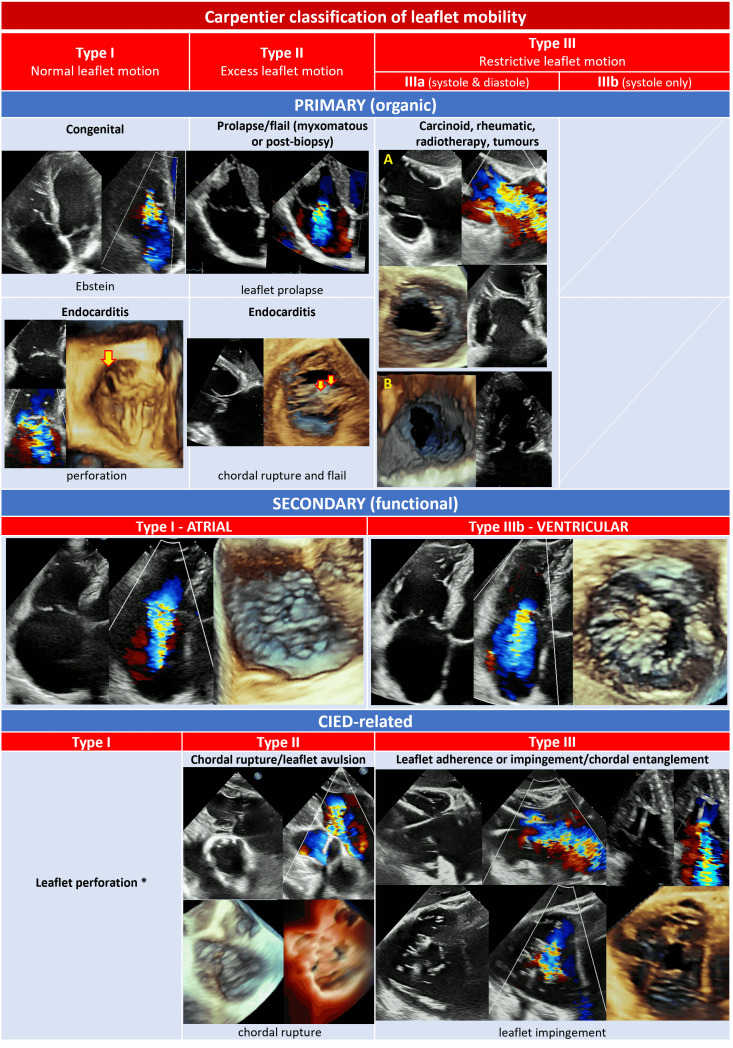

Abbreviations: CIED, cardiac implantable electronic device; A, carcinoid; B, rheumatic. *: rarely reported.

Primary or organic TR is caused by TV abnormalities and accounts for about 8% of TR [57].

Secondary or FTR is a consequence of right heart chambers remodelling, is the most common TR type and is differentiated into ventricular and atriogenic/atrial (or often defined as isolated TR). Ventricular secondary TR is caused by leaflet tethering and papillary muscle displacement, as a consequence of RV dysfunction and enlargement in conditions of volume overload or PH, generally secondary to left heart disease. Atrial secondary TR is characterized by annular dilatation and is related to AF, age, and HF with preserved EF [12, 50, 53]. Right ventricular and RA remodelling patterns in response to different pathological stimuli have been previously described.

Finally, implantation or extraction procedures of CIED in the RV leads may cause significant TR in 7–45% of cases with a plethora of mechanisms: leaflet impingement/adhesion/perforation/avulsion, chordal rupture/entanglement [12, 21]. When evaluating a CIED-related TR, a careful multi-view echocardiographic assessment of TR degree is pivotal, because an underestimation (more frequently with TTE) of the TR severity may occur with colour-Doppler due to acoustic impedance and reflectivity of the CIED leads [21]. 3D datasets of the TV are particularly useful to assess the transvalvular trajectory of the lead and its relationships with leaflets and the subvalvular apparatus. The transvalvular position of RV leads may be commissural, impinging on a leaflet, adherent to a leaflet or in the middle of the valve. Commissural and central trajectories are generally safe and not associated with significant TR. An adherent lead generally moves altogether with the leaflet and may cause TR only if it significantly interferes with the leaflet systolic closure. An impinging leaflet, instead, restricts the leaflet systolic closure and usually causes a significant TR [21].

### 2.2 Cardiac Magnetic Resonance

Echocardiography is the modality of choice for initial work-up of TV disease and is pivotal in guiding TV interventions. However, it has some limitations, mainly due to patient’s body habitus and suboptimal RV visualization also in patients with good acoustic windows.

CMR overcomes all these drawbacks; it has an established role in anatomical and functional assessment of right heart, with a high accuracy and reproducibility in the assessment of right ventricular volumes, right ventricular EF and quantification of TR [58].

#### 2.2.1 CMR Acquisition Protocol

In patients with TR the main goals of CMR are: assessment of right ventricular volume and function, TR grading, anatomical evaluation of venous systemic and pulmonary returns and, finally, tissue characterization. Detailed anatomical study of TV leaflets and mechanism of TR can be done in case of inconclusive echocardiographic studies.

Right chambers remodelling due to TV disease must be assessed with a dedicated CMR protocol that adds some important dedicated sequences to the traditional CMR protocol. The dedicated CMR protocol is summarized in Table 4.

**Table 4.** **CMR protocol for TR**.

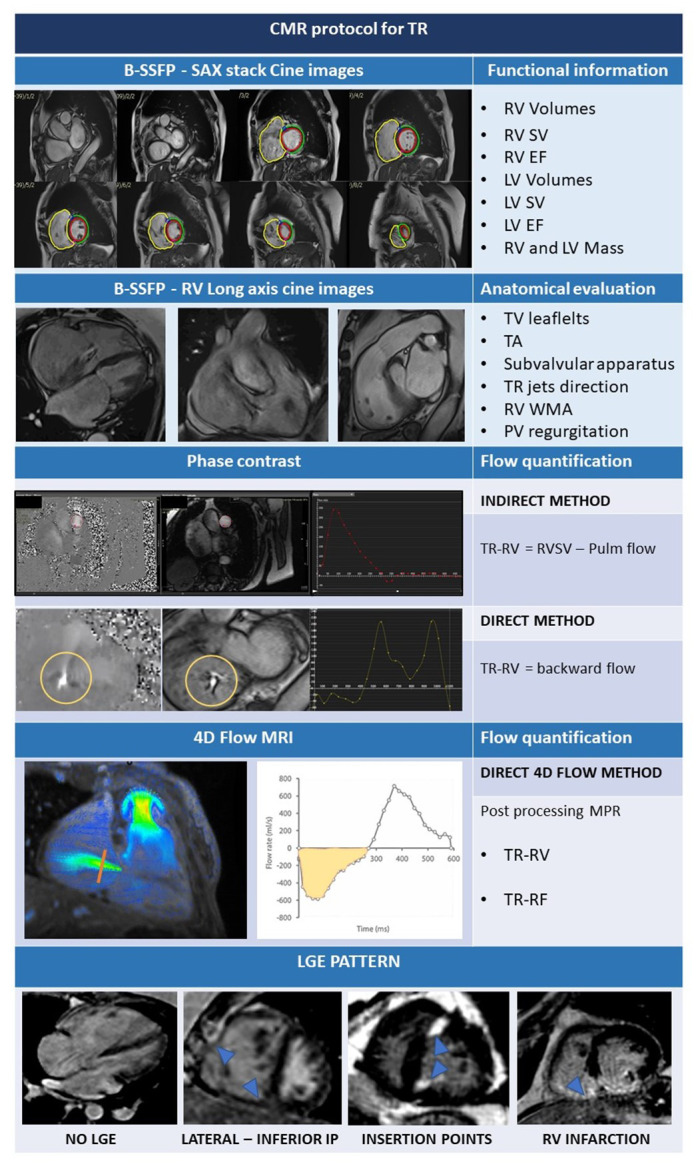

Abbreviations: b-SSFP, balanced steady state free precession; CMR, cardiac magnetic resonance; EF, ejection fraction; IP, insertion points; LGE, late gadolinium enhancement; LV, left ventricle; MPR, multiplanar reconstruction; MRI, magnetic resonance imaging; PV, pulmonary valve; RV, Right ventricle; SV, stroke volume; SAX, short axis; TA, tricuspid annulus; TR, tricuspid regurgitation; TR-RV; tricuspid regurgitation regurgitant volume; TV, tricuspid valve, WMA, wall motion abnormalities.

Balanced steady-state free-precession (b-SSFP) acquisitions have good signal-to-noise ratios and high blood-to-myocardium contrast. Short- and long-axis views can be acquired using b-SSFP sequences. The SAX stuck must be acquired from RV base to apex, taking care to include the most basal part of RV that can be displaced in case of RV enlargement over the plane of LV base.

**Fig. 3.**
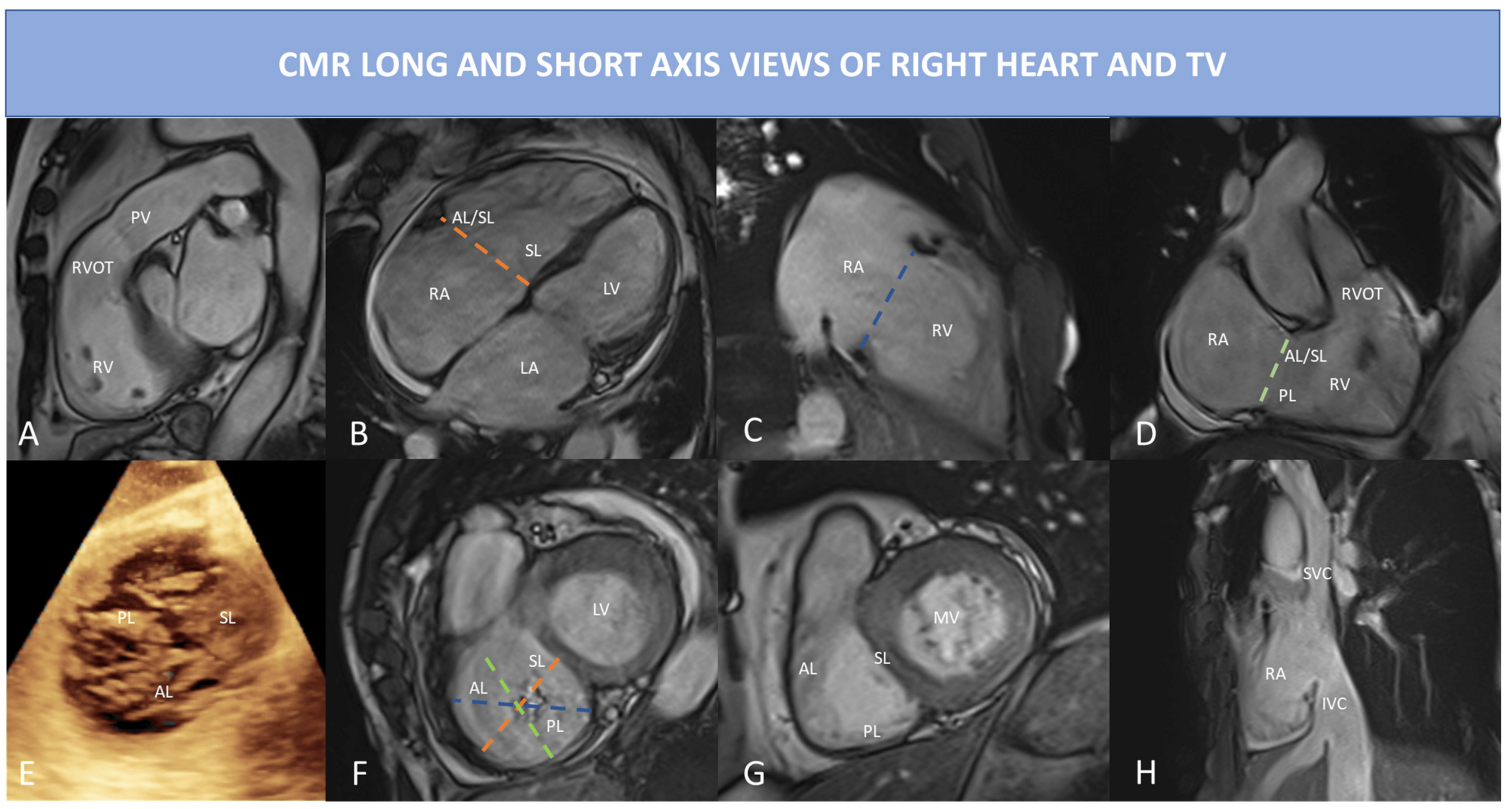
**CMR long and short axis view of right heart and TV using bSSFP sequences**.(A) RVOT view: in this view can be best appreciated pulmonary valve regurgitation and outflow tract. (B) 4 Chamber view: right ventricular inflow, RV and RA are visualized; the dotted orange line indicates the septo-lateral dimension of the TA. (C) RV 2 chamber view or inflow view, only the RA and RV are visualized; the dotted blue line represents the TA. (D) RV inflow/outflow view also called RV 3 chamber view, the dotted light green line shows the antero-posterior dimension of the TA. (E) 3D echocardiography of the TV showing a central coaptation defect in this case of functional TR; all three leaflets are visualized. (F) CMR short-axis systolic view of the TV of the same case of (E): the dotted lines represent the different TA dimension as depicted in (B–D) (but acquired in diastolic phase to measure the largest TA diameters). (G) CMR short-axis diastolic view of TV, the three leaflets are easily identified. (H) bicaval righ atrial view, prescribed using a cutting plane passing through the RA, the IVC and the SVC; in this view both IVC and SVC dimensions can be assessed, the HV is also visualized. Abbreviations: AL, anterior leaflet; b-SSFP, balanced steady state free precession; CMR, cardiac magnetic resonance; HV, hepatic vein; MV, mitral valve; IVC, inferior vena cava; LA, left atrium; LV, left ventricle; PL, posterior leaflet; PV, pulmonary valve; RA, right atrium; RV, Right ventricle; RVOT, right ventricular outflow tract; SL, septal leaflet; SVC, superior vena cava; TA, tricuspid annulus; TV, tricuspid valve.

Additional RV long axis views can be obtained from SAX views, 4 chamber views, coronal and transaxial localizer. These specialized views are: the RV inflow view, the RV inflow/outflow view, and the RV outflow tract (RVOT) view (Table 4 and Fig. 3).

RV transaxial stack can be obtained in a transaxial plane from the level of the diaphragm to the pulmonary bifurcation or as a stack of 4 chamber cine views; it can be useful to identify WMA and TR jets in multiple plains that cannot be visualized in SAX and LAX views. Both RV transaxial stack and SAX stack can be used to calculate RV volumes and function by contouring endocardial borders of RV in systole and diastole from (Table 4). Most of available post-processing software performs automating tracing in few seconds, deriving RV stroke volume (RVSV) and RVEF from RV end-diastolic volume (RVEDV) and RV end-systolic volume (RVESV) calculated using the Simpson’s method without geometric assumption.

After acquisition of b-SSFP, direct flow measurements can be done through phase-contrast sequences. To measure the aortic and pulmonary flow, phase-contrast sequences must be prescribed perpendicular to the LAX of the vessel and the flow direction. Two type of images are than generated: magnitude images and phase velocity maps [59]. Anatomic delineation and contouring of the vessel are made mainly in the magnitude images, while the phase map is used to directly calculate the flow over the cardiac cycle, as it represents the velocities within each pixel of the vessel. TR-regurgitant volume (TR-RV) can be calculated in a direct or indirect way (Table 4). The indirect method is the most used and it integrates information from cine images and phase contrast images by subtracting the forward pulmonary flow obtained by phase velocity maps from the RVSV calculated from the SAX stack (TR-RV mL/beat = RVSV – Pulmonary flow). Once TR-RV is obtained, RF can be calculated as the ratio between TR-RV and RVSV as shown by the formula: RF = TR-RV/RVSV. It is also possible to directly calculate TR by prescribing a phase contrast sequence with a plane parallel to TV annular plane in systole. However, tricuspid annular excursion is usually very wide, TV annular plane is not planar and TR jets often eccentric causing suboptimal evaluation of TR using the direct method.

Recently, volumetric 4D flow MRI has been developed to partially overcome these limitations [60]. 4D flow imaging has several advantages over conventional phase contrast cardiac MRI. The 4D flow can be prescribed as a single volume acquisition covering the entire heart, without the need to prescribe anatomical planes perpendicular to the flow of interest. MPR is easily used in post-processing to visualize regurgitant jets and measure TR-RV. The direct quantification of TR-RV can be done in a single measurement, taking care to measure at least 5 mm apart the valve plane, to avoid regions of velocity-aliasing and segments of severe signal dephasing. Quantification of TR by 4D flow MRI is highly reproducible and consistent across multiple methods of measurement [61]; it also showed excellent interobserver and intraobserver reliability for quantification of TR regurgitant fraction (TR-RF) and TR-RV [62].

Late gadolinium enhancement (LGE) sequences and native T1 mapping are both used for tissue characterization of the right ventricular walls. However, the normal RV wall thickness of 3–5 mm and the low spatial resolution of these sequences limit an adequate analysis of the RV walls. In case of right ventricular transmural infarction or RV walls hypertrophy, the presence of LGE can be easily appreciated, but TR is often accompanied by RV dilatation without hypertrophy due to chronic volume overload. Thus, the RV walls are usually thin and partial volume artifacts can affect RV wall evaluation with both LGE and T1 mapping sequences. Systolic acquisition of LGE sequences can improve RV walls visualization, while high resolution acquisition techniques have been developing and need definite validation [63].

The presence of RV LGE has never been studied in patients with isolated TR, while it has been described in different clinical scenarios. Location and extent of RV LGE may differ according to the underlying disease associated with the TR. Different types of RV LGE are described in Table 4. The most common pattern of RV LGE is the RV insertion point. The clinical relevance of isolated RV insertion point LGE in subjects without additional evidence of cardiac damage and in patients with non-ischemic dilated cardiomyopathy does not convey worse prognosis [64, 65]. There is some evidence that it can be associated with high left ventricular filling pressure and diastolic dysfunction in patients with hypertrophic cardiomyopathy [66]. PH is an important cause of RV walls fibrosis and LGE at the RV junctional insertion points and into the interventricular septum; different patterns have been described with involvement of both RV insertion points and interventricular septum, only one or neither of all [67]. The presence of LGE at RV seems to be more common in case of pre-capillary PH [68]. PH patients with LGE show significantly higher mean PA pressure (obtained with right heart catheterization) and lower RVEF than patients without LGE. In patients with Ebstein’s anomaly LGE can be localized in the RA, in the atrialized RV and in the RV and seems to be associated with the severity of TR and presence of supraventricular arrythmia [69]. In patients with Tetralogy of Fallot (ToF) the presence of LGE relates with higher right ventricular volumes, lower EF and a higher pulmonary regurgitant fraction. In ToF the most common locations of LGE are RVOT, the ventricular insertion points and around the ventricular septal defect patch [70]. In case of a prior RV infarction, transmural LGE is visualized into the RV free wall extending from the inferior LV myocardium or the inferior interventricular septum [71, 72]. In conclusion, the presence of RV LGE is often associated with concomitant LV pathology, PH or ischemic heart disease, however its role in patients undergoing TV intervention is still unknown.

**Fig. 4.**
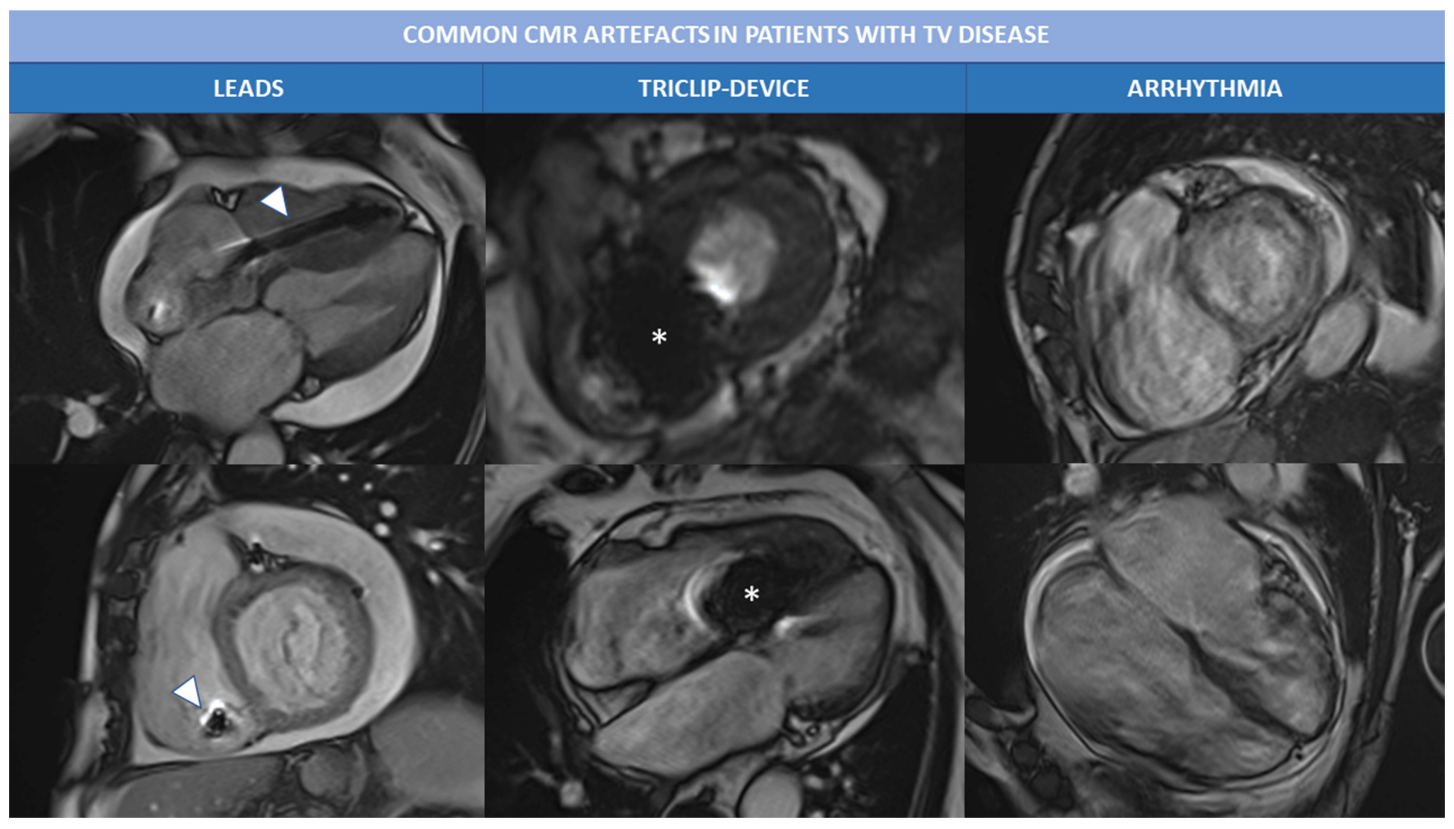
**Common CMR artefacts in patients with TV disease**. Magnetic susceptibility and metallic artefacts are frequent in patients with TR; they are caused by the presence of ferromagnetic components of PM leads (arrowheads) or triclip device (*) and can affect image quality. Arrhythmias, like atrial fibrillation is often associated with severe TR and can cause motion artefacts, as shown in the right column. Abbreviations: CMR, cardiac magnetic resonance; PM, pace maker; TR, tricuspid regurgitation; TV, tricuspid valve.

In patients with TR the exam quality can be affected by the presence of arrythmia (atrial fibrillation is the most common) and/or PM leads or devices (like TriClip). Most common artefacts that occur during Cine bSSFP sequences are presented in Fig. 4. In case of extreme irregular RR intervals, prospective triggering or real-time free-breathing cine sequences can be acquired to avoid artefacts related to HR variability. Leads and other intracardiac devices are responsible of metallic artefacts than can compromise image quality, above all of bSSFP sequences; in this case the use of cine fast-spoiled gradient, instead of bSSFP, can reduce magnetic susceptibility artefacts.

#### 2.2.2 The Right Ventricle and the Right Atrium

Due to the complex shape and structure of RV, multiple CMR planes are required for a comprehensive analysis of right chambers and TV [73].

As previously mentioned, SSFP sequences in RV LAX planes must be part of the acquisition protocol. In the four-chamber view, the trabeculated apex, the moderator band, the inlet, the lateral wall and the interventricular septum are displayed. The inlet, the inlet/outlet and the outlet view are described together with the SAX in Fig. 3.

A change in RV shape and volume can be the first sign of RV dysfunction, pressure or volume overload. In severe TR both signs of pressure and volume overload can coexist according to the underlying disease and TR mechanism.

**Fig. 5.**
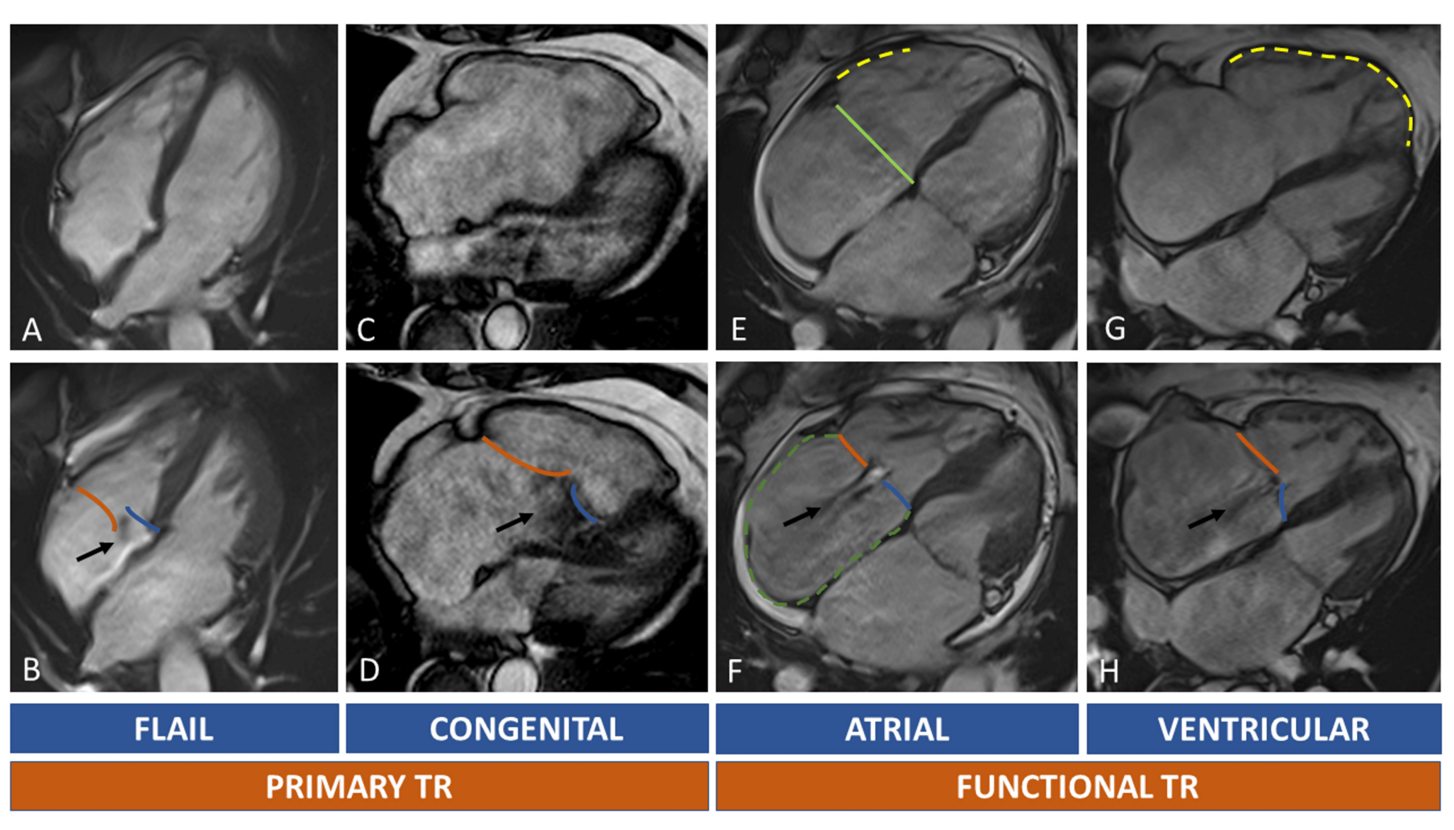
**Tricuspid regurgitation and RH chambers remodelling**. In (A–D) primary TR are shown. Upper row (A,C,E,G) show diastolic images, while the lower row (B,D,F,H) systolic images. The black arrow indicates the TR regurgitant jets. Orange line: anterior/posterior leaflet; blue line: septal leaflet. Light green line: septolateral diameter of TA in diastole. Dotted green line: right atrium. Dotted yellow lines: RV walls dilatation. Primary TR is caused by leaflets abnormalities. A case of flail leaflet is shown in (A,B): right chambers can be normal or mildly dilated because of recent onset of TR; in Ebstein anomaly (C,D) the TR is caused by displacement of septal leaflet (blue line) with distortion of normal right chamber anatomy. Secondary TR can be atrial or ventricular mediated. In atrial functional TR there’s a prevalent enlargement of the basal wall of RV ((E), yellow dotted line), of the TA (light green line) and RA ((F), green dotted line). In ventricular functional TR, the RV is globally enlarged ((G), yellow dotted line) causing leaflets tenting (H). Abbreviations: RA, right atrium; RH, right heart; RV, TA, tricuspid annulus; TR, tricuspid regurgitation; TV, tricuspid valve.

In primary TR, the RA or RV can be normal or mildly dilated during the first phases of the disease; however, as TR begets TR, in long-standing primary TR, signs of volume overload may develop, with RA, RV and TA enlargement. In secondary TR, the mechanism of regurgitation in mainly related to RA or RV dilatation without primary leaflet abnormalities (see Fig. 5).

Atrial and ventricular FTR have different CMR morphological features (Fig. 5). In atrial FTR, there is a severe TA, RA and basal RV dilation. A crucial feature of atrial FTR is the annular dilatation with increase of septum-to-lateral diameter, loss of TA saddle shape and presence of large central coaptation gap of the TV leaflets. This aspect of basal dilatation of RV, without elongation of the lateral wall and the trabeculated apex is called “conical deformation”. Ventricular FTR is usually associated with PH; the mechanism of TR is mainly due to papillary muscles displacement and leaflets tethering secondary to extreme right ventricular apex and lateral wall dilatation, while TA and RA dilation are less evident. In ventricular FTR the morphology of the RV becomes elliptical or spherical with less predominant basal enlargement as seen in atrial FTR [51].

An exhaustive study of RA can be done by prescribing a stack of SAX images that extend over TV plane through the whole RA, as prolongation of SAX stack acquisition of left and RVs [74]. As alternative, RA can be visualized in standard 4 chamber, RV 2 chamber view (inlet view) and inlet/outlet view. RA volume can be calculated both from SAX views or LAX views. However, normal ranges for right atrial volumes vary significantly between methods. The area-length method is used from LAX views, it is faster, but less reproducible than Simpson’s method used with SAX stack.

#### 2.2.3 The TV Annulus and TV Leaflets

Normal TA is oval and has a saddle-shaped structure less evident than that of mitral annulus. Given its non-planar structure it is difficult to visualize using CMR, but its maximum and minimum diameter and its motion can be assessed in SSFP cine sequences with good temporal and spatial resolution (Fig. 3). The two main movement of TA are the base-to apex contraction and the sphincteric contraction. During the sphincteric contraction the anterior and posterior part of the TA move toward the interventricular septum causing a 20–30% reduction in size (diameters, perimeter and area). Thus, TA has its maximum size in late diastole and its minimum in late systole. Measuring TA dimensions is possible both in RV LAX and SAX views (Fig. 3). As alternative, free-breathing whole heart b-SSFP sequences can be acquired both in systole and diastole. The dataset obtained can be analysed with multiplanar reformatting during post-processing. These sequences have the limit of long scan times (7–10 minutes) and the lack of dedicated post-processing software that allow 3D evaluation of TA.

CMR study of TV leaflets may be challenging due to several reasons. First of all, the TV morphology has high interindividual variability; leaflets number can vary from 2 to 5 according to Hahn classification, so that visualization and exact recognition of each leaflet in SAX is more complex and time-consuming than with 3D or 2D echocardiography. In RV LAX views only one or two leaflets can be identified and complex morphology (like type V according to Hahn classification) can be easily missed. Moreover, TV leaflets are usually thin, highly mobile and thus more prone to artifacts and limited characterization due to low temporal resolution. The exact mechanism of a primary TR cannot be fully understood with CMR, and integration with echocardiographic information is of crucial importance. Conversely, in FTR leaflets motion is often reduced because of tethering forces and a reliable measurement of leaflets length, tenting area, coaptation depth and regurgitant orifice area may be possible also with CMR. A reduction of slice thickness of SSFP cine sequences to 5–6 mm without interslice gap may allow a better visualization of both TA and TV leaflets.

#### 2.2.4 TR Severity Quantification

Despite CMR is considered the gold standard for assessment of RV size and function, it has not been validated for the quantification of TR in clinical practice yet [75]. Nevertheless, in the last years the development and technical improvement of CMR have led to increased interest in quantification of valve diseases using non-echocardiographic methods. Many studies showed that CMR assessment of TR is feasible, but less established than that of other regurgitant valvular lesions. CMR quantification methods of TR may be qualitative or quantitative, direct or indirect (Table 5).

**Table 5.** **Contrast injection protocols before TTV**.

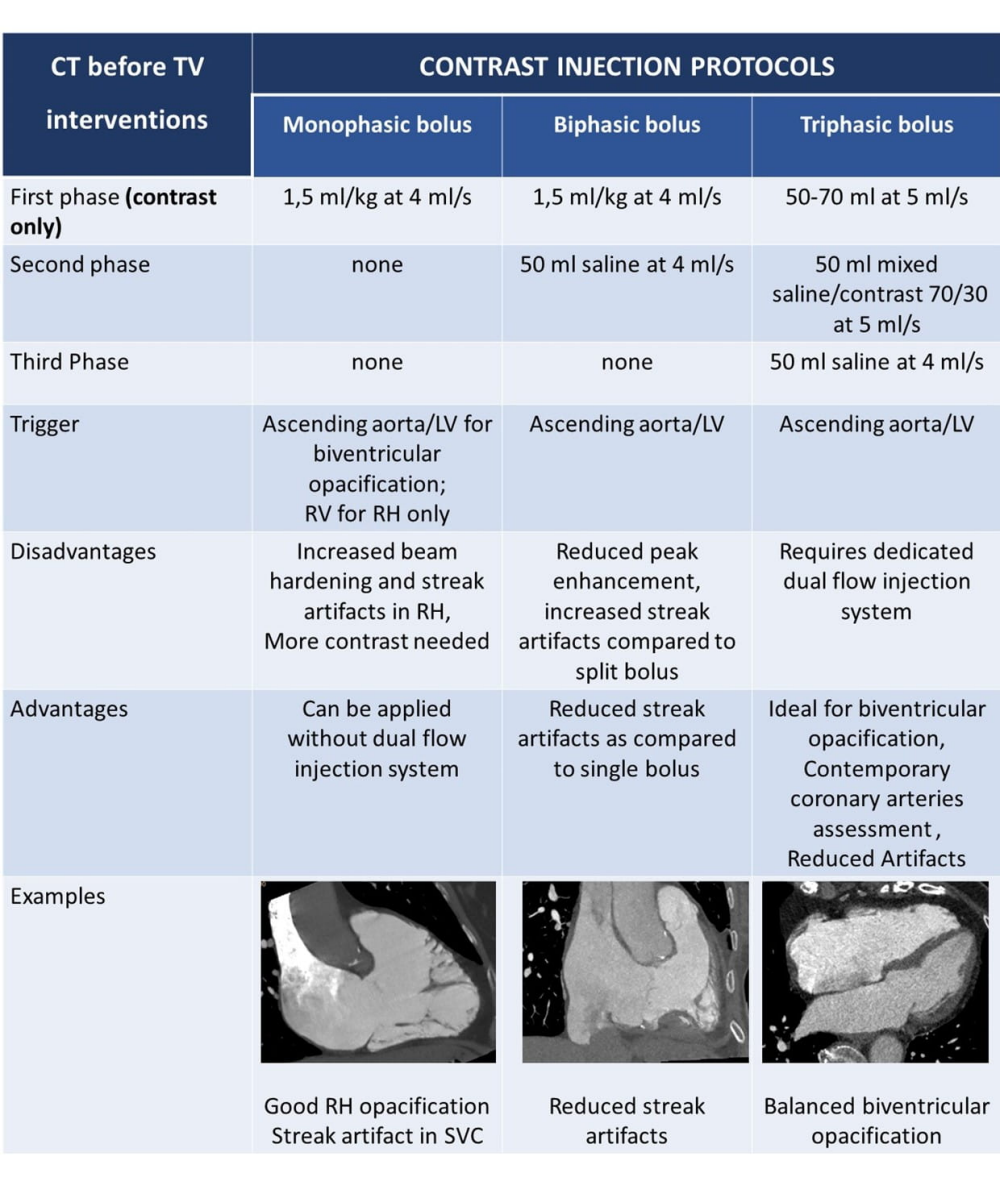

Abbreviations: CT, computed tomography; LV, left ventricle; RH, right heart; RV, right ventricle; SVC, superior vena cava.

**Qualitative assessment**. Qualitative assessment of TR by CMR imaging can be done through bSSFP or gradient echo sequences. The regurgitant jet is visualized as an area of local signal void secondary to flow turbulence or acceleration. In a small study by Reddy *et al*. [76], twenty-nine TR were analysed and the visual assessment of cardiac regurgitant lesion resulted accurate and reproducible with good agreement with standard quantitative assessment. However, a lone visual evaluation is not additive as compared with a more feasible echocardiographic evaluation; moreover, the extent and the intensity of signal voids depend on the sequence used (b-SSFP or gradient echo) and the parameters of the sequence itself (flip angle, echo time, slice thickness, window level). Medvedofsky *et al*. [77] proposed a semi-quantitative approach to TR quantification based on the measure of the size and signal intensity (SI) of the cross-sectional TR jet area in the RA in SAX SSFP images and showed that the jet area significantly increased concurrently with TR severity assessed by echocardiography.

**Quantitative assessment**.The indirect method is the most common used for quantitative assessment of TR. TR-RV is calculated as the difference between RVSV calculated from RVSAX stack and Pulmonary flow derived by phase contrast images. In the 2017 JASE-JCMR guidelines cut-off values for TR severity were based on MR classification and severe TR was defined as having a TRF >48% [14]. Zhan *et al*. [78] validated this hypothesis in a population of 547 patients with FTR, using CMR to quantify regurgitant volume and RF. In this study TR-RV was measured as mentioned above, while TRF was calculated by dividing the TR-RV by the RV inflow, which, in the absence of pulmonary regurgitation is the RVSV. Both TRF and TR-RV were associated with increased mortality after adjustment for clinical and imaging covariates, including RVEF. They identified three different risk categories based on TRF and TR-RV: low risk (TR-RV <30 mL, TRF <30%), intermediate risk (TR-RV 30–44 mL, TRF 30–49%) and severe risk (TR-RV ≥45 mL, TRF ≥50%). The mortality from FTR at 1 year was 15% in those with a TRV of ≥45 mL and 14% in patients with a TRF ≥50% (HR 2,26 and 2,60 respectively) [78].

In a comparison study of 337 patients, echocardiographic parameters of TR severity had variable accuracy against TR-RV by CMR (AUC for VC of 0.65, AUC for EROA of 0.75 and AUC for TR-RV of 0.72). A multiparametric hierarchal approach resulted in 68% agreement with CMR and 100% agreement when a 1-grade difference in TR severity was considered acceptable [79].

Given the large body of evidence concerning echocardiographic quantification and prognostication of TR, the recent CMR data, despite promising, should be used with caution and need to be validated yet in the field of percutaneous treatment of TR.

Direct quantification with phase contrast sequences of atrio-ventricular valves is less validated than PC imaging of semilunar aortic and pulmonic valves. Semilunar valves are more fixed and planar than atrioventricular valves, so that quantification of anterograde and retrograde flow is more reproducible [80, 81]. In a small study, Jun *et al*. [82] reported good agreement of tricuspid flow quantification using direct phase contrast method; however, the published data are very few and this technique is not widespread in clinical practice.

New direct quantification of TR using 4D flow techniques showed high concordance with both direct or indirect methods of quantifying regurgitation [83]. The 4D flow CMR consist of a volumetric, isotropic, time-resolved cine sequence that enables three-directional velocity encoding; post-processing analysis allows calculation of forward flow, reverse flow, regurgitation fraction, and peak velocity. Interestingly, in both children and adult studies comparing indirect quantification to 4D flow, the quantification of TR with 4D flow was more accurate than 4D quantification of MR. Severe eccentric jets are more common in MR, increasing the technical difficulty of maintaining an orthogonal plane to the mitral regurgitant jet, while TR jets are more often central and larger, making easier to find the right cut-plane.

Notwithstanding direct interrogation of the regurgitant jet with 4D flow showed high intra and inter-observer consistency and reproducibility, no data are currently available regarding the prognostic implications of 4D flow derived TR-RV and the specific cut-off values.

### 2.3 Cardiac Computed Tomography

TV surgery is associated with increased mortality, mainly due to patient’s comorbidities and a usual late referral to surgeon. In recent years an increasing number of minimal invasive and transcatheter treatments have been developed. The choice of the optimal transcatheter device therapy needs a careful patient’s selection, mainly based on echocardiographic and CT evaluation. Transcatheter tricuspid valve replacement relies principally on CCT evaluation of the TA, the surrounding structures, the vascular access and right ventricular chambers.

#### 2.3.1 CT Acquisition Protocol

**Patient preparation**: no specific medication is recommended; however, AF is common in this population and thus rhythm irregularity and high heart rate can affect scan quality. Beta-blockers must be used i.v. at the time of the scan or started orally days before. Reasonably an HR <100 bpm should be reached, but an optimal rate of 60–70 bpm can allow contemporary evaluation or coronary arteries provided that nitrates are administered, and an arteriosus phase is acquired. High quality ECG monitoring is essential for a good scan, thus ECG tracing needs to be checked during breath-hold to identify possible artefacts and low tracing signal and eventually improved with lead position change or skin brushing.

**Scanner**: scanner type affects acquisition protocol; if locally available, last generation scanner (at least 64 multidetectors, or dual-source) shall be used to obtain the maximal temporal resolution and to reduce the scan time.

**Coverage**: the acquisition should cover at least a 12–14 cm of the chest, while in case of extreme right/left chamber enlargement even a 16–18 cm coverage is required. Additional non-gated scan can be acquired to cover the venous system and in particular the position of SVC and IVC.

**Slice thickness**: ≤0.75 mm.

**Acquisition**: a gated acquisition from the tracheal bifurcation to just below the diaphragm needs to be planned from traditional scout acquisition of the thorax. The entire cardiac cycle (0–100% of R-R interval) or at least a 10–90% acquisition to cover end-systolic and end-diastolic phases are the preferred acquisition windows. In case of TTVI planning, the scan mode depends mainly on the scanner characteristics and the coverage required. Prospective or retrospective triggering can be used. In case of multidetectors (256–320 detectors rows) a prospective full-volume-1 beat acquisition can be performed. If a less advanced multidetector scanner is available, only a prospective multibeat or a retrospective acquisition are possible; in this case motion and misregistration artifacts are expected. Dual-source scanners allow for high pitch helical scanning with excellent temporal resolution. Non-gated acquisition of venous accesses (from neck to pelvis) can be obtained right after the gated acquisition of the heart and usually do not need further administrations if adequate volume of contrast has been previously used. Tube voltage and current settings should be adapted to patient’s body habitus; in general, 80–120 kV and 400–550 mA are used.

**Contrast administration**: iodinated contrast agents with at least 350 mgI/mL are suggested. Test bolus or bolus tracking techniques are in general chosen based on local expertise. In case of test bolus the region of interest (ROI) must be positioned in the centre of the RV in order to obtain adequate contrast in both RV and venous system. The bolus tracking strategy is a simple and reproducible alternative to test-bolus, ideal in case of step-an-shoot approach without the need for extra-contrast dose administration. Type of contrast injection protocol are summarized in Table 5. A single bolus strategy may not be sufficient for adequate image quality in TTVR planning, while biphasic or split (triphasic) bolus are preferred. Single bolus requires more contrast and lower injection velocities and is associated with frequent beam hardening and mixing artifacts in SVC. When a dual flow injection system is available, triphasic mut be preferred over the biphasic protocol. In triphasic bolus, the first injection is contrast, the second a mixture of contrast/saline solution (30/70 in general), followed by a final saline injection. Flow velocity is set at 4 or 5 mL/sec. This protocol is ideal for bi-ventricular opacification and is associated with lower right heart/SVC streak artifacts compared to both single and dual bolus strategy [84, 85, 86, 87, 88].

**Reconstruction**: we suggest one reconstruction at each 5% of the R-R interval to cover the entire cardiac cycle (0–100% at each 5% of R-R interval).

#### 2.3.2 The Right Chambers

Similarly to CMR, CCT may be used to assess RV heart; its main weaknesses are the radiation exposure, the iodinated contrast medium administration and the lower temporal resolution. The great advantages of CCT are represented by the low scan time, the excellent spatial resolution, and the feasibility in patients with implantable devices. Studies comparing CCT and CMR showed great agreement in terms of volumes and function [89, 90, 91], hence the same threshold values for right chambers quantification could be considered in case of TV disease, together with their prognostic implications. Normal CCT values of right chambers have been derived from studies on healthy subjects [91, 92].

Most of commercially available softwares allow semi-automated contouring of endocardial borders, similarly to CMR. In presence of isolated TR, CCT can be used to calculate TR-RV by subtracting LVSV to RVSV; however, if a concomitant valve regurgitation is present, CCT loses its theoretical utility in valve disease quantification.

#### 2.3.3 The Tricuspid Annulus

The assessment of the elliptical shape of the TA is challenging both with echocardiography (lower spatial resolution than CT, often poor acoustic windows) and with CMR (axial images or a 3D-whole heart acquisition, but with lower spatial resolution than CT).

**Fig. 6.**
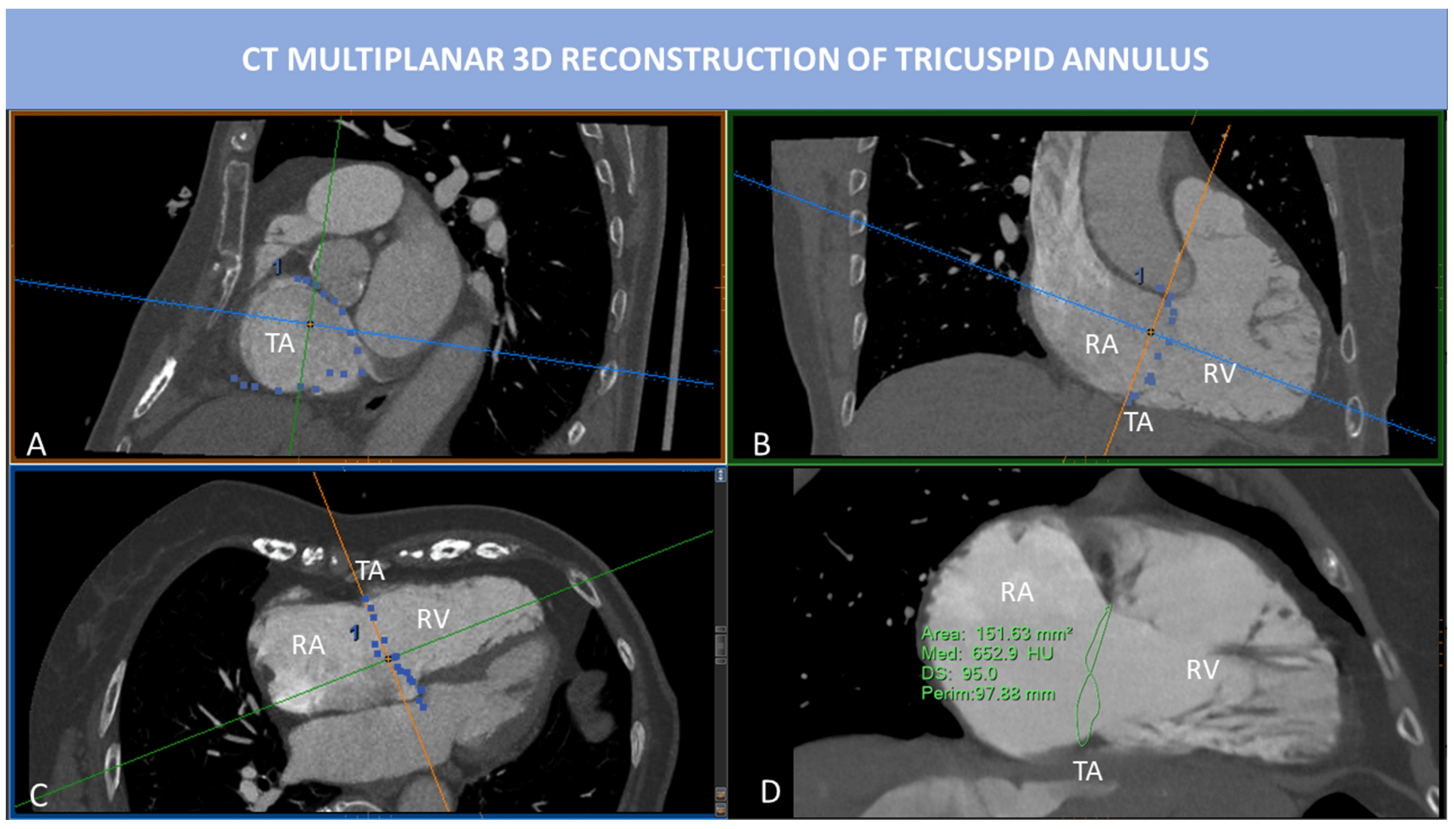
**CT multiplanar 3D reconstruction of TA**. MPR is used to visualize the insertion points of the TV leaflets into the TA. SAX view of TA (A) is obtained by placing two cut-planes passing through TA antero-posterior diameter (B) and septum-to-lateral diameter (C). However, the TA is not planar, and 2D evaluation of TA fails to represent the real shape of TA. In (D), the saddle-shape of TA can be appreciated. 3D TA is obtained tracing point by point the insertions of TV leaflets into the TA. In this way, 3D perimeter and area can be calculated. Abbreviations: CT, computed tomography; MPR, multiplanar reconstruction; RA, right atrium; RV, right ventricle; SAX, short axis; TA, tricuspid annulus; TV, tricuspid valve.

On the other side, CT is able to provide high quality images of TA with adequate temporal resolution and excellent spatial resolution throughout the whole cardiac cycle [93]. A SAX plane at the TA plane can be recreated using MPR with a reliable assessment of anteroposterior and septolateral diameters, perimeter and area both in systole and in diastole. TA can be traced manually or using semi-automated softwares. In a comparison study of Praz *et al*. [94], agreement between TEE and CT for TA sizing resulted superior using semi-automated methods than with direct manual measurements. TA has two main contraction patterns: the sphincteric contraction and the excursion towards the right ventricular apex. Excursion toward the apex can be easily assessed by CMR or echocardiography in 4-chamber view, while sphincteric contraction is best appreciated with CT or 3D TEE echocardiography. A 20 to 30% reduction in size of TA can be seen in systole, so that maximal dimensions are obtained in late-diastole [64]. Normal values of TA are derived by echocardiographic studies using 3D acquisition (end-diastolic maximal circumference and area: 105 ± 12 mm and 860 ± 200 ${\mathrm{mm}}^{2}$, respectively) [95] and a MPR post-processing of 3D dataset. Patients with moderate-to severe and severe TR showed a mean CT-perimeter of 148 ± 16 mm and mean CT-area of 1612 ± 295 ${\mathrm{mm}}^{2}$ in a study of 250 patients by Rosendael *et al*. [96]. In severe TR mean antero-posterior TA diameter was 50.3 ± 5.2 mm, and 41.2 ± 5.6 mm, significantly higher than in patients with mild or moderate TR. In another study, even mild TR showed larger TA dimensions than patients without TR [97]. Given the prevalence of TA dilatation in severe TR, accurate TA sizing is pivotal before TV annuloplasty, valve replacement and transcatheter treatment (Fig. 6).

#### 2.3.4 The Tricuspid Valve Leaflets

Optimal visualization of TV leaflets in CT requires homogeneous contrast medium opacification around the leaflets with a dedicated contrast medium administration protocol (preferably, a triphasic protocol). Even if with a good spatial resolution, CT may show motion artifacts due to high and/or irregular heart rate and excessive leaflets motion, like in case of primary TR (flail, endocarditis). In ventricular FTR the leaflets are tethered and less mobile than in primary TR, thus motion artifacts are generally reduced. Four-chamber and 2-chamber views of the RV may be used to assess the leaflet tethering and accordingly measure leaflet length, tethering angle, tenting eight and tenting area. Compared to patients with moderate (<3+) TR, patients with moderate to severe or severe TR show higher degree of tethering of the anterior and septal tricuspid leaflets, with no differences in terms of tethering angle of posterior leaflet [93]. Interestingly, the tethering height (>7.2 mm) of the TV seems to be associated with the recurrence of TR after TV annuloplasty [98]. Tricuspid anatomical regurgitation orifice area (AROA) can be measured with MPR by contouring the tips of TV leaflets in systole in a similar way to mitral valve regurgitation [99]. The AROA was recently calculated using Dual Source CT in 60 patients with symptomatic TR and compared to TR severity and VCA assessed at TEE. The AROA showed good intra and interobserver reliability and excellent linear correlation with 3D VCA and TR severity [100].

## 3. Pre-Procedural Evaluation before Transcatheter Tricuspid Valve Interventions

### 3.1 Pre-Procedural Echocardiographic Assessment of the Tricuspid Valve for Transcatheter Edge-to-Edge Repair

Several transcatheter techniques and devices have been conceived for the treatment of TR, mainly based on three principles: annuloplasty, leaflet approximation and replacement (orthotopic or heterotopic) [101]. The leaflet approximation or edge-to-edge repair is the most frequently used technique worldwide, currently with two approved devices: TriClip (Abbott Vascular, Santa Clara, CA, USA) and PASCAL (Edwards Lifesciences, Irvine, CA, USA) [12].

Transcatheter edge-to-edge repair (TEER) of the TV presents several anatomical and technical challenges, hence a careful anatomic suitability evaluation, a forward-looking patient selection and an accurate pre-procedural planning of the interventional strategy are fundamental for a successful and prognosis-changing procedure.

Table 6 shows the anatomical and imaging suitability criteria for TEER of the TV. First, a poor or inadequate TEE window is a great hurdle for a TEER procedure; patients without proper TV visibility should not be selected for TEER [12, 102].

**Table 6.** **Anatomical suitability criteria for the TEER of the tricuspid valve**.

	Ideal	Challenging	Unsuitable
Imaging	-	-	Inadequate TEE imaging
Etiology	Limited leaflet prolapse or flail	Presence of CIED leads without leaflet impingement	CIED-related etiology with leaflet impingement
			Leaflet perforation
			Rheumatic or carcinoid (Hedinger syndrome) etiology
			Active endocarditis
Leaflet configuration	Tri-leaflet morphology	Non tri-leaflet morphology	-
Coaptation gap	Antero-septal coaptation gap	Postero-septal or antero-posterior coaptation gap	-
	Coaptation gap <7 mm	Coaptation gap >7 mm but ≤8.5 mm	
Leaflet tethering	-	-	Extreme leaflet tethering
RA anatomy	-	Prominent Eustachian valve or Chiari network	-
		Unfavourable inferior vena cava-right atrium angle	

Abbreviations: CIED, cardiac implantable electronic device; TEE, trans-esophageal echocardiography.

As said before, the definition of TR etiological mechanism is crucial: a rheumatic or carcinoid-related mechanism is generally not suitable for TEER due to the extremely restricted motion and marked thickening of leaflets, with wide “ungraspable” coaptation gaps [12]. An active endocarditis is an obvious contraindication for any transcatheter structural procedure, while a TR due to a healed endocarditic process may be a TEER target, except for leaflet perforations. A CIED-related TR with leaflet impingement is generally deemed unsuitable for TEER.

The variability of leaflet configuration may represent a procedural challenge, as well. As shown by Sugiura *et al*. [103], a TV configuration with more than three leaflets is associated with an increased risk of residual TR, even if more devices are implanted. Possibly, the presence of more commissures may either attenuate the tension force induced by the clips on the septolateral plane or facilitate the onset of new regurgitant gaps along the coaptation lines of supernumerary leaflets, as a consequence of the new distortion forces introduced by the clips.

The coaptation gap size and location are demonstrated predictors of procedural success and post-TEER residual TR. The coaptation gap size is defined as the septolateral dimension of coaptation line between mural and septal leaflets, the most commonly “grasped” coaptation line (only in rare cases the coaptation line between anterior and posterior leaflet is targeted) [103, 104, 105]. While a severe TR with a long gap in antero-posterior direction but with a narrow coaptation gap size may be successfully treated using more devices, a severe TR due to a wide coaptation gap size is difficult to grasp and associated with an increased risk of significant residual TR. Indeed, a large gap (>10 mm) may either impede a successful clip placement or lead to a grasping away from the target region, both resulting in inefficient TR reduction. Moreover, a large coaptation gap size is the expression of a more advanced remodelling of both TV (leaflet tethering and annular dilatation) and right heart chambers. Also, the coaptation gap location has a direct influence on procedural success as a non-central/non-anteroseptal TR jet is associated with increased risk of significant residual TR [103, 104, 105]. A successful grasping in a posterior position is technically challenging, due to an unfavourable angle between IVC and TA. Furthermore, in an experimental ex-vivo model of TEER on FTR, the best hemodynamic results were obtained when grasping was performed between the anterior and septal valve leaflets [106]. As known, tricuspid annular dilation generally occurs towards its antero-lateral side and the anterior leaflet is the largest and the most mobile tricuspid leaflet [17, 107]. Hence, a clip placement in anteroseptal position seems to also have a biomechanical rationale as it employs the overabundant coaptation reserve and mobility of the anterior leaflet, with demonstrated better results [106].

An extreme leaflet tethering is a great challenge for an effective grasping and the expression of an advanced RV remodelling, with an unfavourable patient prognosis. Indeed, Besler *et al*. [105] found that a TV tenting area bigger than 2.1 ${\mathrm{cm}}^{2}$ was a predictor of procedural failure at univariate analysis.

Then, the RA anatomy may interfere with proper positioning of the delivery system, due to a prominent Eustachian valve, a Chiari network, or an unfavourable angle with IVC. In this respect, some authors have proposed the left femoral vein access site of choice for a better delivery system manoeuvrability and to maximize device height from the annular plane [101, 108].

Finally, the ideal TV anatomy any interventionalist would love to treat with a TEER procedure is a confined tricuspid prolapse or flail with a small (<7 mm) antero-septal coaptation gap in a tri-leaflet valve, without CIED, Chiari network or prominent Eustachian valve and with an optimal angle between IVC and RA.

After TEER anatomical suitability is established, the patient selection should enclose several points: symptomatic status, clinical presentation, medical therapy optimization, rhythm control options, end-organ dysfunctions, RV dysfunction severity and PH assessment. The clinical aspects of patient selection go beyond the aim of this manuscript, while the evaluation of RV and pulmonary vasculature diseases are primarily multimodality imaging-based. As suggested by surgical experience, patients with mild or moderate left ventricular impairment, preserved RV function and without evidence of pre-capillary PH may result the best candidate for a prognosis-changing tricuspid TEER. However, the prognostic value of RV systolic function and pulmonary pressure parameters in patients undergoing TTVR is still under investigation. TAPSE and echocardiographic sPAP did not predict clinical outcome after TTVR in the Trivalve registry [109]. On the other hand, a mid-range RV function (TAPSE = 13–17 mm) identified the patient subset in which transcatheter tricuspid intervention was associated with an improved survival in a recent propensity matched analysis [110]. Then, a CMR-derived RVEF ≤45% has shown an independent predictive power of outcomes in a small cohort of patients undergoing tricuspid TEER [111]. The assessment of RV-pulmonary artery coupling might overcome these uncertainties; in particular, an impaired ratio of TAPSE/sPAP (better if invasively derived with a cut-off 0.29 mm/mmHg) and a discordant diagnosis of PH between right heart catheterization and echocardiography (>10 mmHg difference) have shown an independent prognostic power in patients with severe TR undergoing tricuspid TEER [56].

After patient selection and before entering the cath lab, the procedural strategy needs to be planned upfront together by interventionalist and interventional imager, above all when more clips are likely needed. If there is a single or a limited target lesion, the device is generally aimed at the site of the maximum coaptation gap, such as in TEER procedures for mitral valve. However, due to the complexity of TV and the extension of complex-shaped regurgitant area, often the maximum coaptation gap site cannot be directly “grasped” and more than one clip is needed. Several “multiple clip strategies” exist [112, 113]:

- “zipping mode”: when the maximal coaptation gap cannot be directly targeted, the first clip is generally positioned as near as possible in order to reduce the maximal coaptation gap and make it “graspable”;

- “bicuspidalization”: when the maximal coaptation gap involves the anteroseptal coaptation line, clips are placed along this line (between the septal and anterior leaflets), so that the TV becomes a bicuspid valve with a coaptation line between the posterior leaflet and the newly fused antero-septal leaflet;

- “clover” strategy or triple-orifice technique: both antero-septal and postero-septal commissures are targeted with at least one clip between the septal and anterior leaflets and at least another clip between the septal and posterior leaflets. This technique may be used in cases of coaptation gaps involving both anteroseptal and posteroseptal commissures or when the maximal coaptation gap is predominantly posterior, but a “zipping” strategy on the posteroseptal commissure is difficult to realize.

For all TEER strategies, it is strongly suggested to start the procedure with the most anteriorly positioned clip, to avoid shadowing artifacts in the TG views [112].

### 3.2 CMR Key Prognostic Implications before TV Interventions

Untreated severe TR is responsible for increased mortality and HF decompensation. Surgery is recommended in symptomatic patients with severe TR, while can be performed in selected asymptomatic individuals with RV dilatation and/or dysfunction. However, exact threshold and timing of intervention are not established yet. Most data regarding prognosis and surgical indications rely only on echocardiographic evaluation of RV chambers, with many related limitations. Instead, CMR is still underused for patient’s selection before TV surgery or TTVI, even if is considered the gold standard for chamber quantification and definition of RV remodelling and reverse remodelling.

An echocardiographic-based study of 1292 patients with secondary TR demonstrated that RV systolic dysfunction confers worse clinical outcome regardless of the presence of RV dilation [51]. However, only linear echocardiographic dimensions were used (RV basal diameter and TAPSE) that cannot represent the complex geometry of RV muscular fibres contraction. Among 249 patients who underwent TTVR, RV function and sPAP assessed by echocardiography failed to predict clinical outcomes. More recently, Kresoja *et al*. [111] analysed right ventricular contraction patterns using both CMR (also with strain assessment) and echocardiography in a cohort of patients undergoing TTVR. Global RV dysfunction was defined as CMR-derived RVEF <45% and longitudinal RV dysfunction was defined as a TAPSE <17 mm on echocardiography. Patients with a reduction of TAPSE, a preserved radial and circumferential strain and a normal RVEF had most favourable outcomes [111].

A pilot CMR study published in 2010 about patients undergoing TV surgery concluded that CMR RVEDV predicts RV dysfunction after TV surgery, with an indexed RVEDV of 164 mL/$m^{2}$ predictive of a lack of postoperative improvement of RVEF [114]. The relevance of RV chambers quantification in TR patients was than confirmed in a prospective study of patients with planned TV surgery for severe FTR. After correction for covariates, the multivariate analysis confirmed the incremental role of CMR RV EF in the prediction of postoperative cardiac death and major postoperative cardiac events in patients with RVEF <46% [115].

Hinojar *et al*. [116] defined the prognostic value of CMR RV systolic function in TR patients treated with medical therapy alone and specifically that RVEF ≤58%, a RVEDV index ≥100 mL/$m^{2}$ and TRF ≥40% and TR-RV ≥42 mL were predictive of poor prognosis. They also introduced a new parameter, the effective RVEF (eRVEF), calculated as net pulmonary forward flow/RVEDV. eRVEF avoids an overestimation of RVEF in presence of severe TR and showed the strongest association with outcomes, with an incremental value with regards to RVEF and a cut-off point of 34% [116].

As reported in the prior paragraph, natural history of TR was observed in a large cohort of patients who underwent CMR examination; CMR quantification of TR severity using the indirect method, identified patients with the highest mortality risk (TR-RV ≥45 mL, TRF ≥50%) [78].

### 3.3 CCT Prior to TV Interventions

CT scan is pivotal for the pre-procedural planning of TTVI. Transcatheter tricuspid devices can be categorized as: coaptation devices, annuloplasty devices, heterotopic valves, orthotopic prostheses (Table 7A,7B).

**Table 7A.** **CT pre-procedural planning of coaptation and annuloplasy devices**.

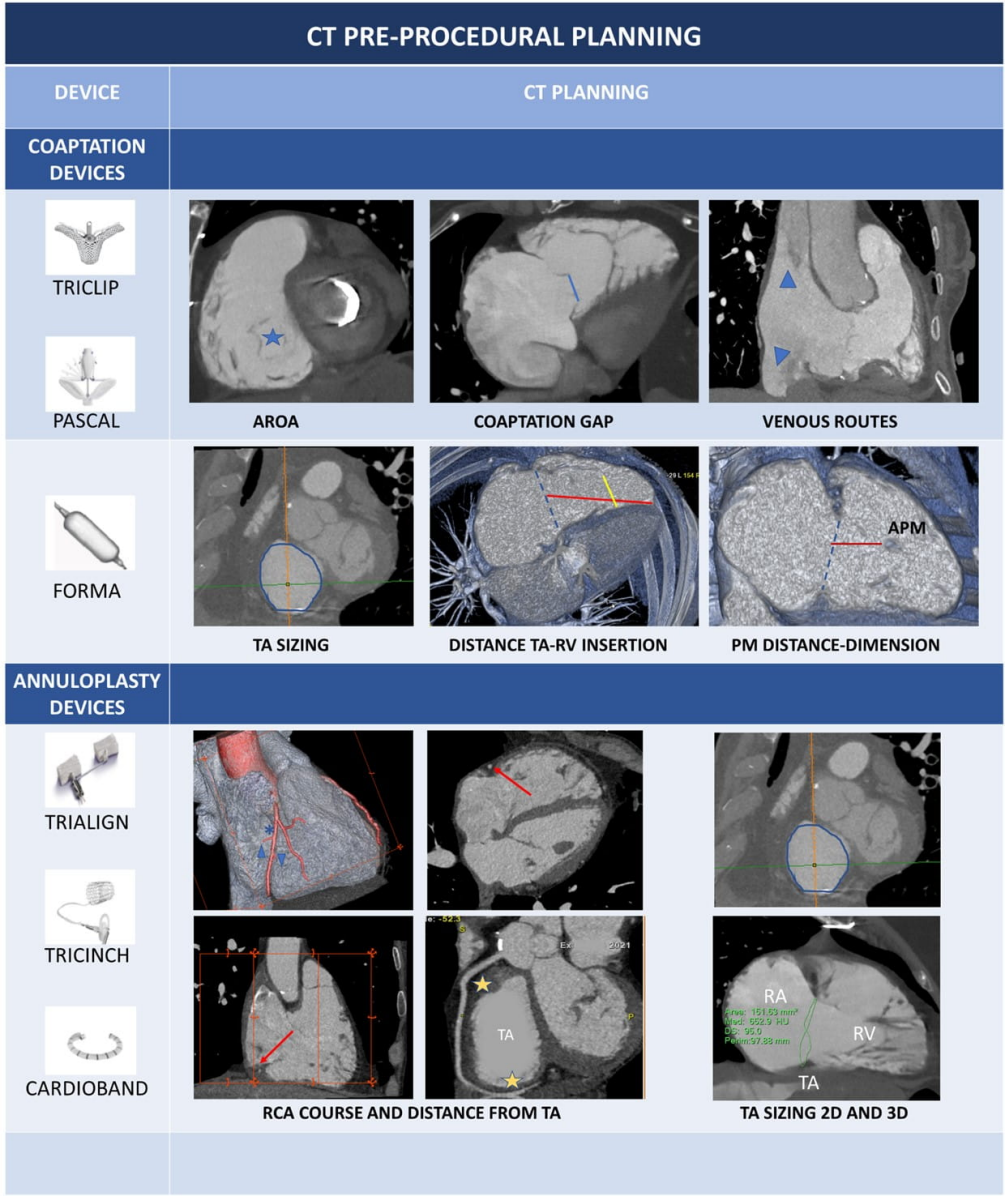

Coaptation devices: Blue star: anatomical regurgitant orifice area (AROA), can be traced from SAX of TV; Blue line: coaptation gap calculated from 4 chamber view; Blue arrowheads indicate inferior and superior vena cava. Forma Device: Continuous circular blue line represents 2D planar contouring of TA; dotted blue line indicates TA; red line is the distance between TA and anchoring point of Forma device to apical septum; yellow line represents the distance between the anterior papillary muscle (APM) and the interventricular septum; purple little line indicates the distance between APM and the TA. Annuloplasty devices: Right coronary artery course and distance from TA must be described before device annuloplasty. RCA course can be in the AV groove (*), superior to TA (up arrowhead) or inferior to TA (down arrowhead). The RCA (in red in the 3D model) is indicated by red arrows in 4 chamber and RV inflow/outflow view. Critical distance points between RCA and TA are highlighted by yellow stars and they refer to proximal and distal RCA, sites of possible injury during the procedure. Abbreviations: APM, anterior papillary muscle; CT, computed tomography; RCA, right coronary artery; RV, right ventricle; TA, tricuspid annulus.

**Table 7B.** **CT pre-procedural planning of eterothopic and orthotopic valves**.

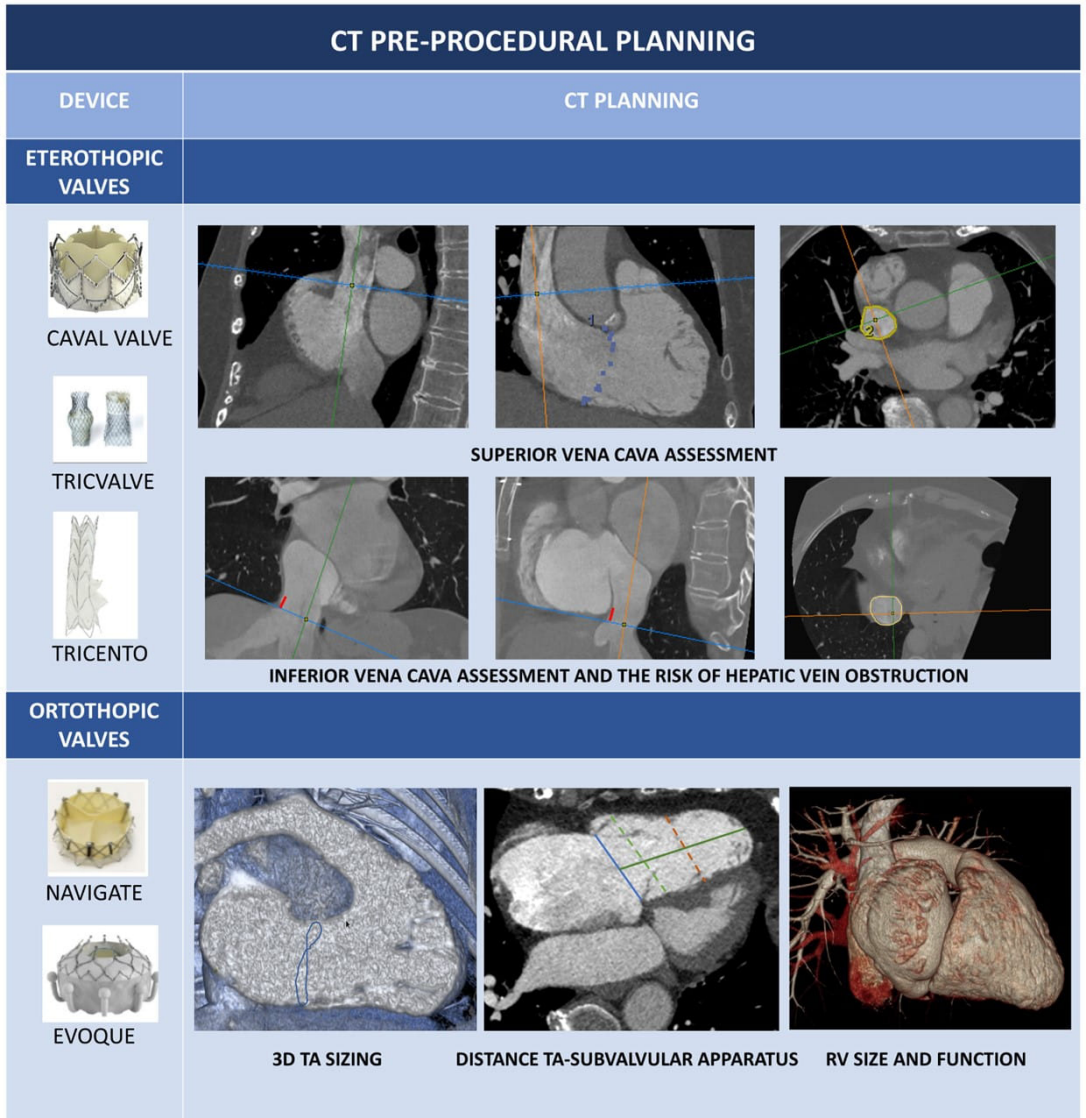

Eterothopic valves: assessment of IVC and SVC is key before eterothopic valves implantation. MPR allows to calculated diameter, area and perimeter (yellow lines) of IVC and SVC. Distance between RA junction and hepatic vein (red line) must be assessed to avoid hepatic vein obstruction. Ortothopic valves: CT 3D dataset MPR allows for careful measurement of distance between the TA and the subvalvular apparatus (moderator band, papillary muscles, trabeculae), the TA and the apex or the TA-RVOT angle and distance. TA dimensions, valvular and subvalvular morphology and vascular access route must also be assessed. Blue line: TA; green line: distance between TA and the apex; green dotted line: basal RV diameter; orange dotted line: mid RV dimension. Abbreviations: CT, computed tomography; IVC, inferior vena cava; MPR, multiplanar reconstruction; RCA, right coronary artery; RV, right ventricle; RVOT, right ventricular outflow tract; SVC, superior vena cava; TA, tricuspid annulus.

#### 3.3.1 Coaptation Devices

Coaptation devices are based on edge-to-edge and edge-spacer-edge valve repair [33, 117]. Patient’s selection for the most commonly available coaptation devices, Triclip (Abbott Vascular, Santa Clara, CA, USA) and Pascal (Edwards Lifesciences, Irvine, CA, USA), rely exclusively on echocardiographic evaluation and CCT is not necessary. However, lead position, leaflets calcifications and the presence of unfavourable angles between IVC and TA can be easily assessed by CCT images during pre-procedural planning. Moreover, in case of inconclusive echocardiographic data, coaptation gap and leaflets length can be measured and TTVI strategy can be changed in case of large coaptation gap or extreme tethering unsuitable for TEER. The Forma spacer device (Edwards Lifesciences, Irvine, CA, USA) is another kind of edge-to spacer device based on a foam-filled polymer balloon that fills the leaflets coaptation gap. The anchoring system is placed in the right ventricular apex, the spacer is placed at the annular level into the coaptation gap, and then the system is then locked in subclavian region. Device sizing is based on coaptation gap and TA dimensions, while the distance between TA and RV apex, as well as between TA and papillary muscles are assessed for device suitability and to avoid anchoring problems (Table 7A). The Forma system requires a large 24F sheath, hence CT scan is useful also to assess adequate dimensions and patency of left subclavian and axillary veins [118].

#### 3.3.2 Annuloplasty Devices

**Fig. 7.**
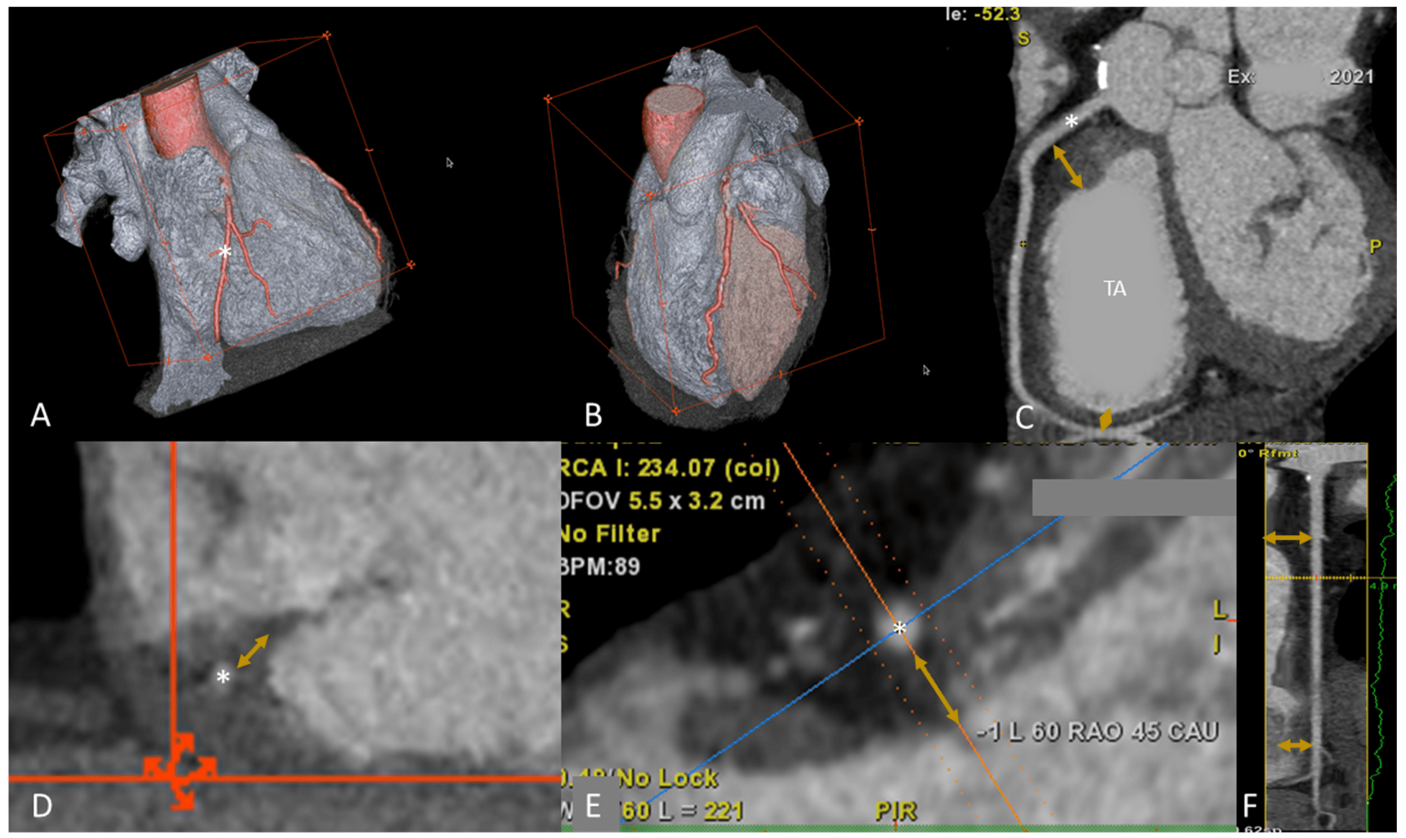
**Right coronary artery evaluation prior to TVI**. With adequate patient preparation and a dedicated triphasic protocol it is possible to evaluate RCA and RH at the same time. (A,B) 3D volume rendering of both right and left chambers; the coronary arteries are highlighted in red, the RCA (*) has a normal course in the AV groove. (C) Relationship between RCA (*) and TA. Distance between proximal and distal RCA (yellow arrows) can be calculated. Proximal or distal RCA of 2 mm, may be associated with high risk of RCA impingement during TV annuloplasty. (D,E) Distance between RCA and TA in LAX views. In (D) the course of RCA is into the AV groove, while in (E) the RCA course is superior the TA. In (F), multiplanar reconstruction algorithm of RCA is shown; the distance between the RCA and the annulus is indicated with orange arrows. Abbreviations: AV, atrioventricular; LAX, long axis; MPR, multiplanar reconstruction; RCA, right coronary artery; RH, right heart; SAX, short axis; TA, tricuspid annulus; TV, tricuspid valve; TVI, tricuspid valve intervention.

The goal of annuloplasty devices is to reduce TA area in FTR. Trialing, TriCinch, Cardioband and Traipta systems are some of the annuloplasty devices currently accessible [119, 120, 121, 122] (Table 7A). Right coronary artery (RCA) complications occur in 15% of patients treated with Cardioband [123] and are considered a major worry in transcatheter annuloplasty. Cardioband anchors are placed in the periannular tissue, and the risk of coronary injury depends on the course and the distance of the RCA around the TA (Fig. 7). The use of a triphasic contrast medium administration protocol may allow to achieve a good visualization of right heart and coronary arteries at the same time. In a study of 250 patients with TR who underwent CT evaluation, the course of RCA had 3 main configurations: along the TA (65% of patients), superior to TA (10%) and crossing the TV (25%). Distance between RCA and TA was measured in mid-diastole, using either a SAX view of the TA in case of RCA running at the same level or a long axis view in case of superior or inferior course. The authors suggest that a maximal distance between the anterior or posterior part of the TA (at the level of the anterior or posterior leaflet insertion) and the proximal or distal RCA of 2 mm, may be associated with high risk of RCA impingement [111]. In the TriCinch device, a corkscrew delivery system is advanced through femoral access to the target region of TA, where the coil is implanted. The target zone is the antero-posterior commissure of TA (at 9–10 o’clock with a ventricular en-face view of TV); at this level, the distance between TA and RCA must be assessed. Among the first 18 patients implanted, RCA injury occurred in one patient. Instead, the goal of the Trialign system is the bicuspidalization of TV, obtained by positioning two pledgets in the septo-posterior and antero-posterior commissures; the two pledgets are then approximated to reduce annular size and regurgitant orifice area. In the first trial, RCA damage requiring stenting occurred in about 6% of cases, with the proximal RCA near to the antero-posterior commissure being the area at higher risk. Finally, the Transatrial intrapericardial tricuspid annuloplasty (TRAIPTA) consists of an indirect annuloplasty repair thorough a loop delivered along the atrioventricular groove within the pericardial space, which is reached by puncturing of right atrial appendage (RAA). Relationship between RAA and the surrounding structures must be evaluated during pre-operative CT planning.

#### 3.3.3 Heterotopic Valves

An alternative approach to direct intervention to TV is the heterotopic implantation of valves in the caval veins. The aim of this approach is to reduce the systemic congestion that characterize patients with severe untreatable TR and right HF. After a first attempt to adapt transcatheter aortic valve prostheses to cava veins, specific devices have been developed.

The TricValve system (P&F Products and Features, Vienna, Austria) consists of two dedicated self-expanding valves, implanted in the superior and inferior cava veins; the Tricento device (NVT AG, Muri, Switzerland) consist of a bicavally anchored covered stent with a lateral bicuspid porcine valve, implanted from top (SVC) to IVC. Role of CT is crucial to assess IVC and SVC dimensions. The SVC must be measured at the level of the innominate vein confluence, of the pulmonary artery and of SVC-RA junction; the IVC must be measured at the level of IVC-RA junction, at the confluence of the hepatic veins and at 5 cm below IVC-RA shift. To avoid hepatic vein obstruction, a distance >10 mm from the RA-IVC junction must be ensured [124, 125] (Table 7B).

#### 3.3.4 Orthotopic Valves

The complex anatomy of TV and the great variety of mechanisms leading to TR make TTVI challenging, and many patients result non suitable for TEER of the TV. Theoretically a transcatheter tricuspid valve prosthesis should be ideal for this non-operable subset of patients; however, many technical issues still remain to be solved. A first limitation is based on the large TA dimensions that require larger prostheses than usual, causing problems in venous access routes and delivery systems management. Secondly, the TV is usually a non-calcified valve with thin and degenerated leaflets, that reduce prosthetic valve stability. Third, TA is very mobile, has very wide systolic excursions and it has extremely variable size during the cardiac cycle, complicating accurate prosthetic sizing. Moreover, the septal leaflet is part of the Koch triangle that is in close relationship with the atrioventricular node, thus a prosthesis implantation may lead to permanent conduction disturbance and the need for a pacemaker implantation.

A great variety of prostheses has been developed for the TV, with a great variability of pre-procedural planning CT protocols. In most cases each device has a specific acquisition protocol, and the images are then analysed by the manufacturer’s Core-lab.

Assessment of TA dimensions, valvular and subvalvular morphology and vascular access routes, are only some of the aspects of the pre-procedural CT imaging and prosthesis sizing. The MPR of CT 3D dataset allows a careful measurement of distance between the TA and the subvalvular apparatus (moderator band, papillary muscles, trabeculae) and between the TA and the apex, and the TA-RVOT angle and distance (Table 7B). Given the large variety in shape of the new TV prostheses, all these parameters must to be taken into account to choose the optimal implantation depth and to reduce the risk of procedural complications (for example RVOT obstruction) [126, 127, 128].

Strength and weakness of each imaging modality are summarized in Table 8.

**Table 8.** **Strength and weakness of each imaging modality in the work-up of tricuspid valve disease**.

	Echo	CMR	CT
Anatomical assessment			
TV leaflets	+++	+	+
TV annulus	++	+	+++
RH chambers	++	+++	+++
Surrounding structures	+	++	+++
Functional assessment			
TR mechanism	+++	++	+
TR severity	+++	+++	+
RV function	++	+++	++
TTVI procedural planning			
TEER	+++	+	+
Other Device Therapies	++	+	+++

Each modality is judged as follows: sufficient (+), good (++) and excellent (+++). Abbreviations: CMR, cardiac magnetic resonance; CT, computed tomography; RH, right heart; RV, right ventricle; TEER, transcatheter edge-to-edge repair; TR, tricuspid regurgitation; TTVI, transcatheter tricuspid valve intervention; TV, tricuspid valve.

## 4. Intra-Procedural Guidance during TV Transcatheter Edge-to-Edge Repair

The intra-procedural guidance of TEER of the TV with TriClip device is based mainly on TEE and fluoroscopy. Hence, the pre-procedural screening requires a careful check of good-quality imaging of the key TEE views.

The main intraprocedural TEE windows are ME, DE and TG windows with a constant use of 3D and biplane modalities [43]. The quality of TEE imaging may be suboptimal in many cases due to the unfavourable anterior position of the TV, which may cause several shadowing artifacts (lipomatous atrial septum, atrial septal defect closure devices, mitral or aortic valve calcium or prosthetic valves), or to an inadequate individual echocardiographic window, which may be further deteriorated by the supine position in the cath-lab [69]. If there is image deterioration due to supine position, placing a towel or pillow under the right shoulder of the patient may improve the image quality.

**Fig. 8.**
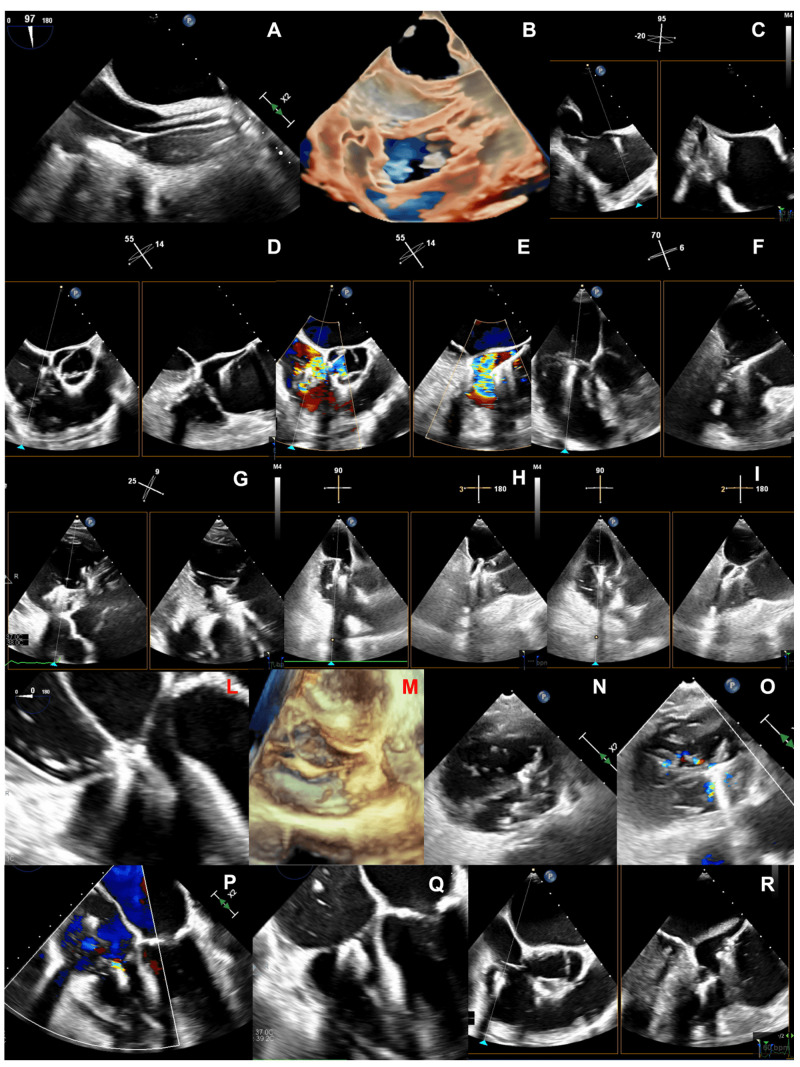
**Steps of intraprocedural imaging**. (A,B) Insertion of the steerable guide catheter into the right atrium. (C–E) Advancement of the clip delivery system through the steerable guide catheter into the right atrium and steering towards tricuspid valve plane. (F) Axial alignment of the clip delivery system. (G) Clip rotation and alignment to the coaptation line. (H,I) Leaflet grasping. (L–N) Check of adequate leaflet tissue grasping. (O,P) Assessment of residual TR. (Q) Clip deployment. (R) advancement of a second clip into the right atrium.

Here follow the procedural steps of TEER for TV, altogether with the main required TEE views (Fig. 8) [43, 59, 129].

(1) Insertion of the steerable guide catheter into the RA (Fig. 8A,B): ME bicaval or modified bicaval (with TV in sight), TG LAX with entry point of IVC into RA in the view, 3D view of RA.

(2) Advancement of the clip delivery system through the steerable guide catheter into the RA and steering towards TV plane (Fig. 8C–E): same views of the first step plus the inflow-outflow view (or intercommissural view) with biplane (or cross-plane) mode for the steering manoeuvre towards the target lesion. The interaction of the clip delivery system and the clip itself with the interatrial septum have to be carefully followed to avoid a perforation of the interatrial septum. As the basic principle of TEER for TV is to approximate the mural leaflet to the septal one, which works as an anchor, the inflow-outflow view (ME or DE) is fundamental because, together with the biplane-derived 4 chamber views, it allows to entirely span the septal leaflet and to guide the clip in anterior-posterior and septal-lateral directions towards the target lesion.

(3) Axial alignment of the clip delivery system (Fig. 8F): ME or DE inflow-outflow views with biplane mode, TG views. The trajectory of the clip delivery system towards the target lesion should be properly adjusted so that the clip results perpendicular to the TV plane.

(4) Clip rotation and alignment to the coaptation line (Fig. 8G): TG with biplane mode (simultaneous assessment of SAX and LAX).

(5) Leaflet grasping (Fig. 8H,I): ME or DE inflow-outflow views with biplane mode with the primary plane positioned perpendicular to the clip arms showing the clip position along the coaptation line. As already mentioned, the inflow-outflow view is crucial for a successful tricuspid TEER procedure as it permits a quick and effective localization of the clip along the entire coaptation line with the septal leaflet.

(6) Check of adequate leaflet tissue grasping (Fig. 8L–N): TG with biplane mode (simultaneous assessment of SAX and LAX), ME or DE inflow-outflow views with biplane mode, ME 4-chamber and DE 2-chamber views, 3D imaging from any window and with additional use of real-time MPRs. The quality check of leaflet grasp is based on several factors: restriction of leaflet motion and clip stability on 2D imaging, adequate tissue bridge on 3D imaging, and TR reduction with colour-Doppler.

(7) Assessment of residual TR (Fig. 8O,P) and trans-valvular gradient (<4 mmHg): ME or DE inflow-outflow views with biplane mode, ME 4-chamber and DE 2-chamber views, 3D imaging for vena contracta area (VCA) analysis which is particularly useful in case of multiple residual regurgitant orifices.

(8) Clip deployment (Fig. 8Q): views of the previous step. After clip deployment a reassessment of clip stability and residual TR is necessary; the clip delivery system is then safely withdrawn under echocardiographic guidance unless another clip is needed (Fig. 8R).

Differently from typical guiding protocols, the so-called “Mainz-Approach”—developed by da Rocha e Silva *et al*. [69]—is based on the TG views as the primary imaging planes to guide almost entirely the TEER procedure of the TV, even the procedural steps of clip introduction into RV and the leaflet grasping. Finally, also fluoroscopy has a role in guiding the TEER procedure, mainly through two perpendicular views: the left anterior oblique caudal view, which coincides with the TG SAX of the TV or a 3D en-face view, and the right oblique caudal view, corresponding with a TG LAX 2-chamber view [130].

## 5. Conclusions

TV disease represents a major health problem that affects a wide proportion of HF patients. Traditional surgery is not often applicable due to high mortality and multiple comorbidities. TTVI offer an extraordinary and promising possibility to treat these patients with a great and increasing number of available devices. A tailored approach for each patient is needed to ensure the best procedural and clinical results and to improve their survival and quality of life. Multimodality imaging is the key of the selection and planning processes. Heart failure specialists, imagers and interventional cardiologists have to be aware of the strengths and limitations of each modality and should learn how to integrate them into a comprehensive and tailored diagnostic and therapeutic pathway. Only the right imaging modality for the right patient and the right device will give the best procedural and prognostic results.

## Abbreviations

2D, two-dimensional; 3D, three-dimensional; AF, atrial fibrillation; AROA, anatomical regurgitation orifice area; (b)-SSFP, (balanced) steady-state free-precession; (C)CT, (cardiac) computed tomography; CIED, cardiac implantable electronic device; CMR, cardiac magnetic resonance; DE, deep-esophageal; DT, deep-transgastric; EF, ejection fraction; EROA, effective regurgitant orifice area; FTR, functional tricuspid regurgitation; HF, heart failure; IVC , inferior vena cava; LAX, long axis; LGE, late gadolinium enhancement; ME, mid-esophageal; MPR, multiplanar reconstruction; PH, pulmonary hypertension; RA, right atrium; RAA, right atrial appendage; RCA, right coronary artery; ROI, region of interest; RV, right ventricle; RVEDV, right ventricle end-diastolic volume; RVEF, right ventricle ejection fraction; RVESV, right ventricle end-systolic volume; RVOT, right ventricle outflow tract; RVSV, right ventricle stroke volume; SAX, short axis; sPAP, systolic pulmonary artery pressure; SVC, superior vena cava; TA, tricuspid annulus; TAPSE, tricuspid annular plane excursion; TEE, trans-esophageal echocardiography; TEER, transcatheter edge-to-edge repair; TG, trans-gastric; ToF, Tetralogy of Fallot; TR, tricuspid regurgitation; TRAIPTA, transatrial intrapericardial tricuspid annuloplasty; TR-RF, tricuspid regurgitation-regurgitant fraction; TR-RV, tricuspid regurgitation-regurgitant volume; TTE, trans-thoracic echocardiography; TTVI, transcatheter tricuspid valve intervention; TTVR, transcatheter tricuspid valve repair; TV, tricuspid valve; VC, vena contrasta; VCA, vena contracta area; WMA(s), wall motion abnormality(ies).
